# Aberrant TGF-β1 signaling activation by MAF underlies pathological lens growth in high myopia

**DOI:** 10.1038/s41467-021-22041-2

**Published:** 2021-04-08

**Authors:** Xiangjia Zhu, Yu Du, Dan Li, Jie Xu, Qingfeng Wu, Wenwen He, Keke Zhang, Jie Zhu, Linying Guo, Ming Qi, Ailin Liu, Jiao Qi, Guangyu Wang, Jiaqi Meng, Zhenglin Yang, Kang Zhang, Yi Lu

**Affiliations:** 1 Eye Institute, Eye & ENT Hospital, Fudan University, Shanghai, China; 2 NHC Key Laboratory of Myopia (Fudan University); Key Laboratory of Myopia, Chinese Academy of Medical Sciences, Shanghai, China; 3 Shanghai Key Laboratory of Visual Impairment and Restoration, Shanghai, China; 4 Beijing Institute of Ophthalmology, Beijing Tongren Hospital, Capital Medical University, Beijing, China; 5 Center for Biomedicine and Innovations, Faculty of Medicine, Macau University of Science and Technology and University Hospital, Macau, China; 6 Guangzhou KangRui Biological Pharmaceutical Technology Company, Guangzhou, China; 7 Institute of Genetics and Developmental Biology, Chinese Academy of Sciences, Beijing, China; 8 Guangzhou Women and Children’s Medical Center, Guangzhou Medical University, Guangzhou, China; 9 Department of Radiology, Eye & ENT Hospital, Fudan University, Shanghai, China; 10 Center for Biomedical Imaging, Fudan University, Shanghai, China; 11 State Key Laboratory of Medical Neurobiology and MOE Frontiers Center for Brain Science, Fudan University, Shanghai, China; 12 Sichuan Provincial Key Laboratory for Human Disease Gene Study and the Center for Medical Genetics, Department of Laboratory Medicine, Sichuan Academy of Medical Sciences & Sichuan Provincial People’s Hospital, University of Electronic Science and Technology, Chengdu, China; 13 Research Unit for Blindness Prevention of Chinese Academy of Medical Sciences (2019RU026), Sichuan Academy of Medical Sciences & Sichuan Provincial People’s Hospital, Chengdu, Sichuan China; 14 Guangzhou HuiBoRui Biological Pharmaceutical Technology Co., Ltd, Guangzhou, China

**Keywords:** Hereditary eye disease, Lens diseases, Refractive errors, Retinal diseases

## Abstract

High myopia is a leading cause of blindness worldwide. Myopia progression may lead to pathological changes of lens and affect the outcome of lens surgery, but the underlying mechanism remains unclear. Here, we find an increased lens size in highly myopic eyes associated with up-regulation of β/γ-crystallin expressions. Similar findings are replicated in two independent mouse models of high myopia. Mechanistic studies show that the transcription factor MAF plays an essential role in up-regulating β/γ-crystallins in high myopia, by direct activation of the crystallin gene promoters and by activation of TGF-β1-Smad signaling. Our results establish lens morphological and molecular changes as a characteristic feature of high myopia, and point to the dysregulation of the MAF-TGF-β1-crystallin axis as an underlying mechanism, providing an insight for therapeutic interventions.

## Abstract (web summary)

High myopia is associated with lens changes, but the underlying mechanisms are unclear. Here, the authors show increased equatorial diameter of the lens in subjects affected by high myopia, and find that these changes are associated with an increase in crystallin expression driven by the transcription factor MAF and TGF-β1 signaling.

## Introduction

High myopia, defined as spherical equivalent ≥−6.00 diopters or axial length ≥26 mm, is a leading cause of blindness worldwide, estimated to affect 9.8% of the global population in 2050^1,2^. Lens, as a transparent ellipsoid organ located in the anterior segment of the eye, is the core refracting medium that is responsible for the full range of vision^3^. Patients with high myopia seem to have higher rate of lens diseases^4,5^. Yet despite previous reports of various mechanisms in myopia progression^6–9^, lens changes in highly myopic eyes and the underlying molecular pathogenesis remain largely unknown.

Aberrant growth of lens in high myopia may be one of the important pathological changes that induce a series of perioperative problems of lens replacement surgery, such as intraocular lens malposition, and affect the surgical outcomes^10,11^. However, previous studies showed no difference in lens thickness between highly myopic and emmetropic (non-myopic) eyes^12,13^. Hence we speculated that the lens diameter of highly myopic eyes might be larger, which was not reported before. In this study, we provide the first solid evidence of larger equatorial diameter of lens in highly myopic eyes by assessment of lens dimensions using magnetic resonance imaging (MRI) in large samples, corroborating our hypothesis.

Larger lens requires increased production and accumulation of structural proteins, of which 90% are crystallins that mainly consist of three families of α-, β-, and γ- crystallins^14^. In particular, β/γ-crystallins (including CRYBA1, CRYBA2, CRYBA4, CRYBB1, CRYBB2, CRYBB3, CRYGA, CRYGB, CRYGC, CRYGD, CRYGN, CRYGS, etc.) together compose over 77% of the total crystallins^15,16^. As previous studies have demonstrated a decreasing trend of α-crystallins, a type of small heat shock proteins, in the lens of highly myopic eyes^17,18^, it stands to reason that the larger lens in high myopia might be related to changes of β/γ-crystallins.

In light of the above hypothesis, we conducted a microarray analysis of human lens epithelium collected from subjects receiving lens surgery. As expected, we detected and verified a significant increase of β/γ-crystallins in lens of highly myopic eyes, which was positively correlated with the lens size. The increased lens size and β/γ-crystallin expressions were recapitulated in two independent myopic mouse models: defocus-induced myopic mouse model and interphotoreceptor retinoid-binding protein (*Irbp*) knockout (KO) spontaneous myopic mouse model. Further mechanistic studies revealed an essential role for transcription factor MAF, in regulating β/γ-crystallin gene expressions by its direct binding to their promoters and by activation of downstream TGF-β1-Smad signaling, which together increase the crystallin production in the highly myopic lens.

## Results

### Pathological lens growth was identified in human with high myopia

**Fig. 1:**
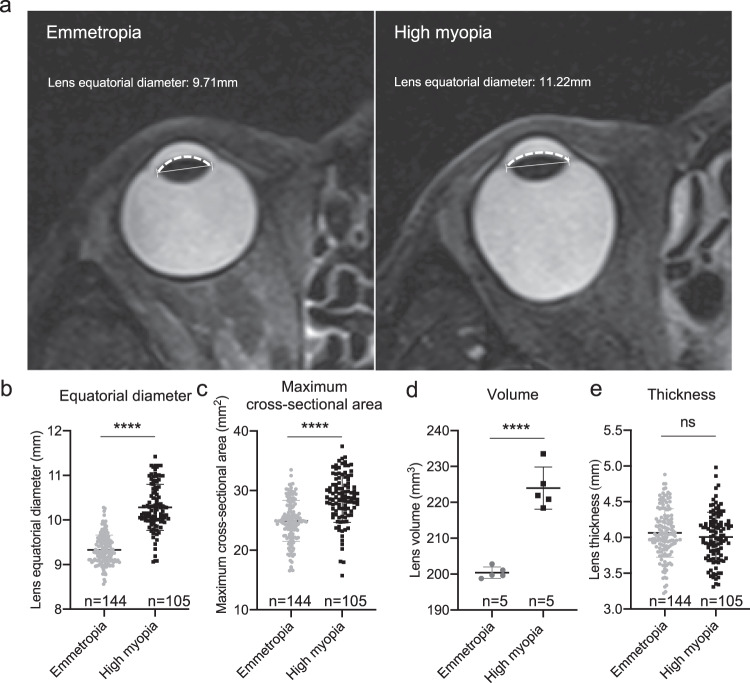
Pathological lens growth was identified in human with high myopia. **a** Representative magnetic resonance imaging of an emmetropic control eye and a highly myopic eye. The capped lines represent the equatorial diameter of lens. The dotted curves represent the anterior surface of lens showing an obviously flatter lens in highly myopic eyes. **b** Lens equatorial diameter (*n* = 144 in emmetropic group vs. 105 in highly myopic group, *p* = 1.39e−45). **c** Maximum cross-sectional area of lens (*n* = 144 in emmetropic group vs. 105 in highly myopic group, *p* = 7.35e−14). **d** Lens volume (*n* = 5 in emmetropic group vs. 5 in highly myopic group, *p* = 2.42e−5). **e** Lens thickness (*n* = 144 in emmetropic group vs. 105 in highly myopic group, *p* = 0.208). Results are expressed as mean ± SD. Level of significance was detected using two-sided Student’s *t* test (**b**–**e**). ****p < 0.0001 and ns represents no significant difference. Source data are provided as a Source Data file.

To test the hypothesis of the bigger lens in high myopia, ocular MRI data of 105 highly myopic eyes and 144 emmetropic eyes were analyzed (Fig. 1a). The two groups were comparable in age (49.36 ± 14.49 vs. 50.63 ± 14.33 in the control) and sex (42.9% male vs. 45.1% in the control). Axial lengths were 28.04 ± 1.68 mm vs. 23.40 ± 0.59 mm in the control. Lens of highly myopic eyes had larger equatorial diameter (10.28 ± 0.52 vs. 9.33 ± 0.33 mm in control), maximum cross-sectional area (28.69 ± 4.01 vs. 24.92 ± 3.45 mm^2^ in control), and larger lens volume upon 3D reconstruction (223.99 ± 5.87 vs. 200.40 ± 1.59 mm^3^ in control) (*p* = 1.39e-45, *p* = 7.35e-14 and *p* = 2.42e-5, Fig. 1b–d). As for lens thickness, no significant difference was seen between the two groups (Fig. 1e). With significantly larger equatorial diameter and similar thickness, the anterior surface of the lens in highly myopic eyes was obviously flatter than that of emmetropic control eyes (Fig. 1a).

### Up-regulation of β/γ-crystallins in lens of highly myopic subjects

**Fig. 2:**
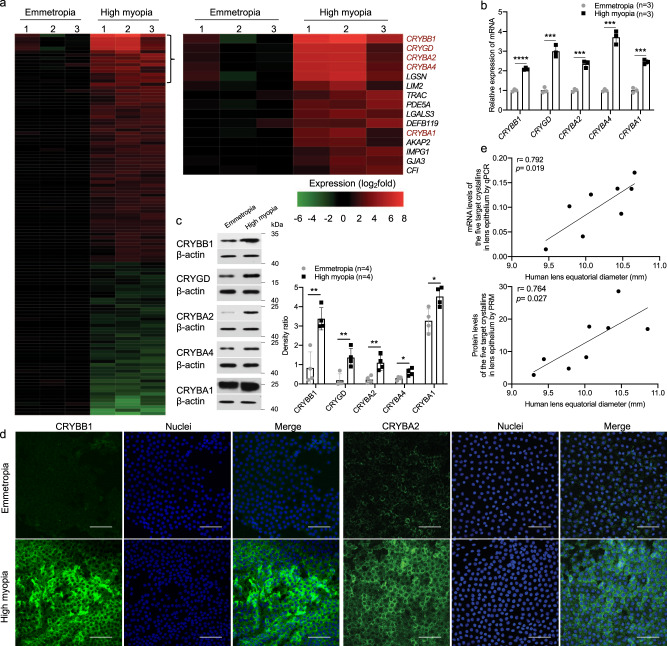
Significant up-regulation of β/γ-crystallins was found in human with high myopia. **a** Heat map from gene expression microarray analysis of up-regulated and down-regulated genes in lens epithelium of emmetropic control and highly myopic eyes showing 117 differentially expressed genes (*n* = 3 vs. 3, fold change ≥ 2, all *p* < 0.05, *CRYBB1 p* = 0.009, *CRYGD p* = 0.032, *CRYBA2 p* = 0.024, *CRYBA4 p* = 0.022, *CRYBA1 p* = 0.032). Right, the top 15 up-regulated genes. **b** qPCR analyses of mRNA expression of the five β/γ-crystallin genes (*n* = 3, *CRYBB1 p* = 2.11e−5, *CRYGD p* = 0.0005, *CRYBA2 p* = 0.0003, *CRYBA4 p* = 0.0002, *CRYBA1 p* = 0.0001). **c** Examination of protein expression by Western blotting of the five β/γ-crystallin genes (*n* = 4, CRYBB1 *p* = 0.002, CRYGD *p* = 0.007, CRYBA2 *p* = 0.007, CRYBA4 *p* = 0.021, CRYBA1 *p* = 0.019). Right, the band density was normalized to loading control as a ratio for statistical analysis. **d** Immunofluorescence images of CRYBB1 and CRYBA2 staining in the human lens epithelium (*n* = 7). Scale bar: 50 μm. **e** Correlation between the increased mRNA and protein levels of the five target β/γ-crystallins in lens epithelium and the lens equatorial diameter in highly myopic eyes (examined by qPCR and PRM). *n*=biological replicates. In (**a**) and (**d**), one specimen from an individual was used as one sample; in (**b**, **c**, and **e**), pooled samples were used, of which in (**e**), lens epithelial samples were pooled in order of lens equatorial diameter (three pieces of lens epithelial samples pooled as one and totally 48 pieces used here) and the average lens equatorial diameter of each three pooled samples were used in the scatterplot. Results are presented as mean ± SD. Level of significance was detected using two-sided Student’s *t* test (**a**–**c**). Associations between variables were evaluated by Pearson correlation analysis (**e**), two-sided test was used). ****p < 0.0001, ***p < 0.001, **p < 0.01, and *p < 0.05. Source data are provided as a Source Data file.

After confirming the larger lens in highly myopic eyes, gene expression profile was examined in lens epithelium from three highly myopic eyes and three emmetropic control eyes using a microarray method. There were totally 70 up-regulated genes and 47 down-regulated genes in the highly myopic group (fold change ≥ 2 plus *p*-value < 0.05). Among the 70 up-regulated genes, eight were genes encoding structural proteins of lens and the top four and 11th were members of β/γ-crystallin genes (in the order of fold change: *CRYBB1*, *CRYGD*, *CRYBA2*, *CRYBA4, and CRYBA1*; Fig. 2a). Up-regulation of the five β/γ-crystallin members in high myopia was validated using pooled human lens epithelial samples (3–6 specimens per assay, 3–7 replicates) by qPCR (Fig. 2b), Western blotting (Fig. 2c) and immunofluorescence staining (Fig. 2d), while no other crystallins were found significantly up-regulated (Supplementary Fig 1a). The expression levels of the five β/γ-crystallins in lens epithelium were positively correlated with an increase in the lens equatorial diameter in highly myopic eyes (Fig. 2e: *r* = 0.792 and *p* = 0.019 at an mRNA level by qPCR, and *r* = 0.764 and *p* = 0.027 at a protein level by parallel reaction monitoring (PRM)).

In addition to the findings in the lens epithelium, we investigated the abunduance of different crystallins in lens mass by PRM and identified an increase of CRYBB1, CRYGD, and CRYBA1 proteins in highly myopic patients (Supplementary Fig 1b).

### Phenotype confirmation of a large lens size and increased β/γ-crystallins levels in two mouse models of high myopia

To re-confirm our findings in highly myopic subjects, lens from two mouse models of high myopia: defocus-induced myopic mice and *Irbp* KO spontaneous myopic mice, were studied.

**Fig. 3:**
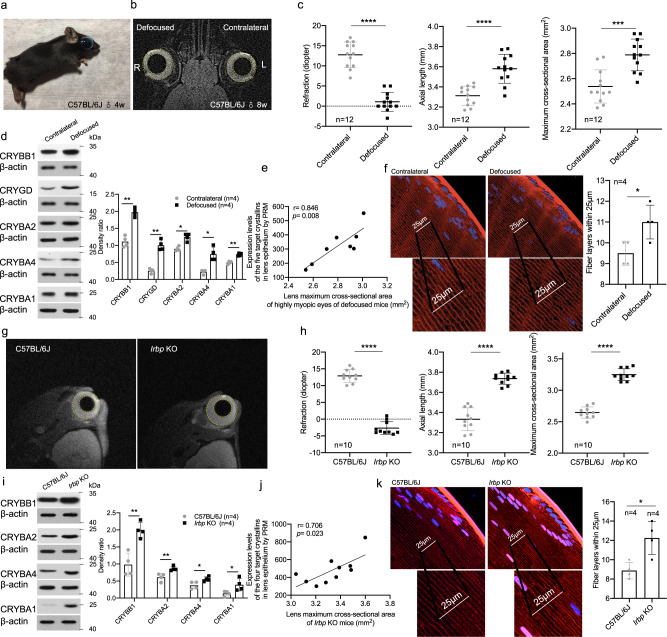
Increased lens size and β/γ-crystallin expression in two mouse models of high myopia. **a** A representative photo of a mouse wearing −25*D* lens to induce high myopia in the right eye. **b** Representative MRI image (coronal image) of an 8-week old mouse with defocus-induced high myopia in the right eye. Dotted yellow lines indicate the contour of the eyeball and the lens. **c** The refraction, axial length and maximum cross-sectional area of mouse lens (*n* = 12, *p* = 1.36e−7, *p* = 3.23e−5, and *p* = 0.0006, respectively). **d** Western blotting of protein expression of crystallin genes in lens epithelium of defocus-induced highly myopic eyes (*n* = 4, CRYBB1 *p* = 0.005, CRYGD *p* = 0.002, CRYBA2 *p* = 0.030, CRYBA4 *p* = 0.028, CRYBA1 *p* = 0.009). Right, the band density was normalized to loading control as a ratio for statistical analysis. **e** Correlation between the increased protein levels of five target crystallins in lens epithelium and the maximum cross-sectional area of lens from highly myopic eyes of defocused mice (examined by PRM). **f** Wheat germ agglutinin (WGA) staining of lens fiber layers located within a length of 25 μm approximately 50 μm beneath the lens surface at the equator (*n* = 4, *p* = 0.014). **g** Representative MRI images (sagittal images) of 8-week wild type mice and 8-week interphotoreceptor retinoid-binding protein (*Irbp*) knockout (KO) mice. Dotted yellow lines indicate the contour of the eyeball and the lens. **h** The refraction, axial length and maximum cross-sectional area of mouse lens (*n* = 10, *p* = 5.64e−13, *p* = 7.73e−9, and *p* = 1.86e−11). **i** Western blotting of protein expression of crystallin genes in lens epithelium of *Irbp* KO mice and C57BL/6J mice (*n* = 4, CRYBB1 *p* = 0.003, CRYBA2 *p* = 0.008, CRYBA4 *p* = 0.030, CRYBA1 *p* = 0.044). Right, the band density was normalized to loading control as a ratio for statistical analysis. **j** Correlation between the increased protein levels of four target crystallins in lens epithelium and the maximum cross-sectional area of lens from *Irbp* KO mice (examined by PRM). **k** WGA staining of secondary lens fiber layers located within a length of 25 μm approximately 50 μm beneath the lens surface at the equator (*n* = 4 in each group, *p* = 0.012). *n*=biological replicates. In (**d**, **e**, **i**, and **j**), pooled samples were used, of which in (**e** and **j**), lens epithelial samples were pooled in order of maximum cross-sectional area (five pieces of lens epithelial samples pooled as one and totally 40 defocus-induced highly myopic mice and 50 *Irbp* KO mice used here) and the average lens maximum cross-sectional area of each five pooled samples were used in the scatterplot. Results are expressed as mean ± SD. Level of significance was detected using two-sided paired *t* test (**c**, **d**, and **f**), and two-sided Student’s *t* test (**h**, **i**, and **k**). Association between variables was evaluated by Pearson correlation analysis (**e** and **j**, two-sided test was used). ****p < 0.0001, ***p < 0.001, **p < 0.01, and *p < 0.05. Source data are provided as a Source Data file.

For the defocus-induced myopic mice, male C57BL/6J mice (4 weeks of age) were subjected to continuous defocus (−25.00D lens) to the right eye (Fig. 3a). Mice with ≥6.00D of myopia at 8 weeks into the defocused eye (vs. the left eye, as evaluated using an infrared photorefractor) were used for MRI (Fig. 3b) and further experiments. The refraction of the defocused eyes was more myopic (1.08 ± 2.35 D in the defocused eyes vs. 12.75 ± 3.19 D in the contralateral eyes, *p* = 1.36e−7, Fig. 3c) and the axial length was longer (3.58 ± 0.14 mm in the defocused eyes vs. 3.31 ± 0.09 mm in the control eyes, *p* = 3.28e−5, Fig. 3c). Maximum cross-sectional area of lens was also larger in the defocus-induced highly myopic right eye (2.79 ± 0.13 in the defocused eyes vs. 2.54 ± 0.13 mm^2^ in the control eyes, *p* = 0.0006, Fig. 3c). Similarly, gene expression profile was examined using microarray in lens epithelium from defocus-induced highly myopic mice. Among the 14 crystallin genes that human and mouse have in common, mRNA levels of *Crybb1, Crygd, Cryba2, Cryba4, Crybb3, Cryga, and Crygs* were significantly up-regulated in myopic eyes of defocus-induced highly myopic mice compared to the contralateral control eyes (*n* = 4 vs. 4, Supplementary Fig 2a). Similar to the findings in human lens epithelium, higher protein levels of CRYBB1, CRYGD, CRYBA2, CRYBA4, and CRYBA1 were also detected in lens epithelium of defocus-induced highly myopic eyes of C57BL/6J mice by Western blotting (Fig. 3d). Further analysis by PRM showed increased protein levels of the five β/γ-crystallins in mouse lens epithelium were also positively correlated with the maximum cross-sectional area of the lens in the defocused eyes (*r* = 0.846 and *p* = 0.008, Fig. 3e). Immunofluorescence staining by wheat germ agglutinin (WGA) which binds to the glycoprotein of the cell membrane showed greater fiber compaction- significantly more lens fiber layers in the highly myopic eyes around the equator in the germinative zone vs. the control eyes (11.0 ± 0.8 vs. 9.5 ± 0.6, *p* = 0.014, Fig. 3f).

Furthermore, eyes of *Irbp* KO mice were also highly myopic (−2.68 ± 1.94 D in *Irbp* KO mice vs. 12.91 ± 1.92 D in wild type mice, *p* = 5.64e−13) with a significantly longer axial length (3.74 ± 0.06 in *Irbp* KO mice vs. 3.33 ± 0.11 mm in wild type, *p* = 7.73e−9) and larger maximum cross-sectional area of lens (3.25 ± 0.09 in *Irbp* KO mice vs. 2.65 ± 0.09 mm^2^, *p* = 1.86e−11) (Fig. 3g, h). In addition, the gene expression profiling of lens epithelium from *Irbp* KO mice showed that of the 14 genes that human and mouse have in common, *Crybb1, Crygd, Cryba4, Cryba1, Crybb2, and Cryga* were significantly up-regulated compared to wild type C57BL/6J mice (*n* = 3 vs. 4, Supplementary Fig 2b). Likewise, up-regulated protein levels of CRYBB1, CRYBA2, CRYBA4, and CRYBA1 (four of the five up-regulated β/γ-crystallins found in human lens epithelium), were identified in the lens epithelium of *Irbp* KO mice by Western blotting (Fig. 3i). A further analysis by PRM showed increased expression levels of these four β/γ-crystallins in mouse lens epithelium were positively correlated with the maximum cross-sectional area of the lens in highly myopic eyes of *Irbp* KO mice (*r* = 0.706 and *p* = 0.023, Fig. 3j). Immunofluorescence staining by WGA also showed greater fiber compaction in which there were significantly more secondary lens fiber layers in the highly myopic eyes around the equator in the germinative zone vs. the control eyes of wild type mice (12.3 ± 1.7 vs. 8.9 ± 0.9, *p* = 0.012, Fig. 3k).

### MAF directly up-regulated lens β/γ-crystallin expression in high myopia

**Fig. 4:**
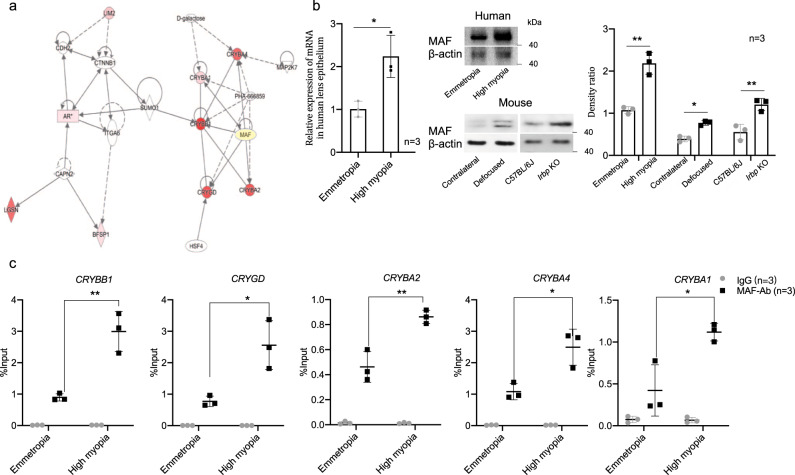
Elevated MAF with higher recruitment to β/γ-crystallin gene promoters in high myopia. **a** The self-defined network established by Ingenuity Pathway Analysis. **b** Examination of mRNA level of *MAF* in human lens epithelium by qPCR analyses (*n* = 3, *p* = 0.015) and protein levels of MAF in lens epithelium of human and mouse highly myopic eyes by Western blotting (*n* = 3, *p* = 0.002, *p* = 0.023, and *p* = 0.008, respectively). Right, the band density was normalized to loading control as a ratio for statistical analysis. **c** ChIP-qPCR analyses of the recruitment of MAF to *CRYBB1*, *CRYGD*, *CRYBA2*, *CRYBA4*, and *CRYBA1* promoters in lens epithelium from emmetropic and highly myopic patients (*n* = 3, *p* = 0.005, *p* = 0.018, *p* = 0.007, *p* = 0.018, and *p* = 0.020, respectively). IgG was used as a negative control and the data presented were mean values relative to input (input%). *n*=biological replicates. In (**b** and **c**), pooled samples were used. Results are expressed as mean ± SD. Level of significance was detected by two-sided Student’s *t* test (emmetropia vs. high myopia and C57BL/6J vs. *Irbp* KO in (**b** and **c**)) and two-sided paired *t* test (contralateral vs. defocused in (**b**)). **p < 0.01, and *p < 0.05. Source data are provided as a Source Data file.

To find the upstream regulator of lens β/γ-crystallins up-regulation in the highly myopic lens, an Ingenuity Pathway Analysis was conducted and suggested the transcription factor MAF may play an important role in directly interacting with the five target β/γ-crystallin genes and their activation (Fig. 4a)^19,20^. Using pooled human lens epithelial samples, we confirmed an increased MAF expression at both mRNA and protein levels in the highly myopic lens (Fig. 4b). Higher expression of MAF was also verified in the lens of two mouse models of high myopia by Western blotting (using pooled mouse lens epithelial samples, Fig. 4b).

To further investigate a possible role of MAF in the regulation of the five β/γ-crystallins, ChIP-qPCR analysis was then conducted. Increased recruitment of MAF to the promoters of *CRYBB1*, *CRYGD*, *CRYBA2*, *CRYBA4,* and *CRYBA1* in lens epithelium of human highly myopic eyes was revealed (Fig. 4c).

**Fig. 5:**
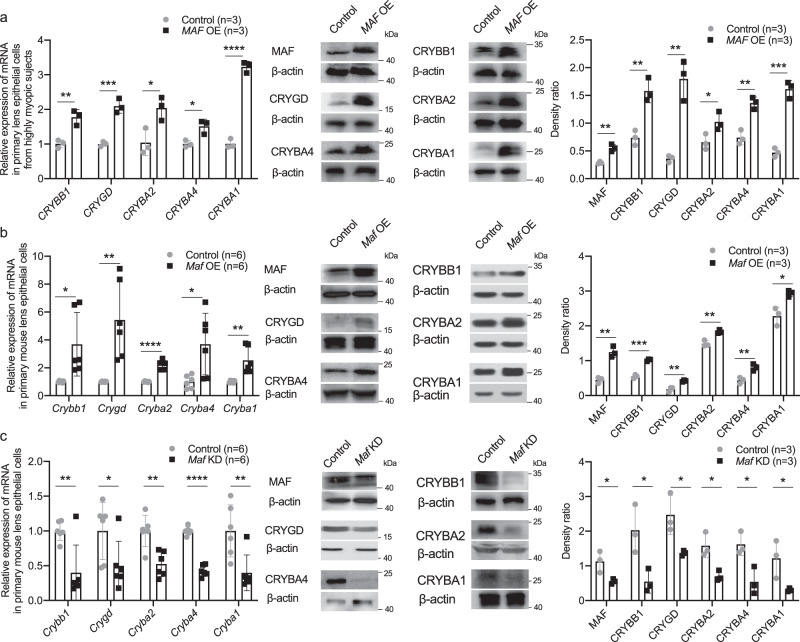
MAF directly up-regulated β/γ-crystallin expression in high myopia conditions. **a** Changes of β/γ-crystallin gene expression detected by qPCR (*n* = 3, *CRYBB1 p* = 0.006, *CRYGD p* = 9.86e-4, *CRYBA2 p* = 0.026, *CRYBA4 p* = 0.017, *CRYBA1 p* = 4.93e−5) and Western blotting (*n* = 3, MAF *p* = 0.002, CRYBB1 *p* = 0.004, CRYGD *p* = 0.002, CRYBA2 *p* = 0.044, CRYBA4 *p* = 0.003, CRYBA1 *p* = 0.0003) in primary human LECs collected from highly myopic eyes after treatment of *MAF* overexpression (*MAF* OE). Right, the band density in Western blotting was normalized to loading control as a ratio for statistical analysis. **b** Changes of crystallin gene expression in primary mouse LECs detected by qPCR (*n* = 6, *Crybb1 p* = 0.016, *Crygd p* = 0.003, *Cryba2 p* = 2.45e−6, *Cryba4 p* = 0.015, *Cryba1 p* = 0.003) and Western blotting (*n* = 3, MAF *p* = 0.002, CRYBB1 *p* = 0.0002, CRYGD *p* = 0.005, CRYBA2 *p* = 0.004, CRYBA4 *p* = 0.007, CRYBA1 *p* = 0.015) after treatment of *Maf* overexpression. Right, the band density was normalized to loading control as a ratio for statistical analysis. **c** Changes of crystallin gene expression in primary mouse LECs detected by qPCR (*n* = 6, *Crybb1 p* = 0.006, *Crygd p* = 0.048, *Cryba2 p* = 0.002, *Cryba4 p* = 5.87e−8, *Cryba1 p* = 0.009) and Western blotting (*n* = 3, MAF *p* = 0.035, CRYBB1 *p* = 0.029, CRYGD *p* = 0.028, CRYBA2 *p* = 0.016, CRYBA4 *p* = 0.022, CRYBA1 *p* = 0.044) after treatment of *Maf* knockdown (KD). Right, the band density was normalized to loading control as a ratio for statistical analysis. n=biological replicates. Results are expressed as mean ± SD. Level of significance was detected using two-sided Student’s *t* test (**a**–**c**). ****p < 0.0001, ***p < 0.001, **p < 0.01, and *p < 0.05. Source data are provided as a Source Data file.

In primary cultured lens epithelial cells (LECs) from highly myopic subjects, *MAF* overexpression using plasmid transfection increased the mRNA level and protein level of *CRYBB1, CRYGD, CRYBA2, CRYBA4,* and *CRYBA1* (Fig. 5a). In primary mouse LECs, over-expressing *Maf* using plasmid transfection increased both the mRNA level and protein level of *Crybb1*, *Crygd*, *Cryba2*, *Cryba4,* and *Cryba1* (Fig. 5b), while knockdown of *Maf* using siRNA transfection induced the opposite effects (Fig. 5c).

### TGF-β1-Smad signaling is activated in lens of high myopia in an autocrine manner

**Fig. 6:**
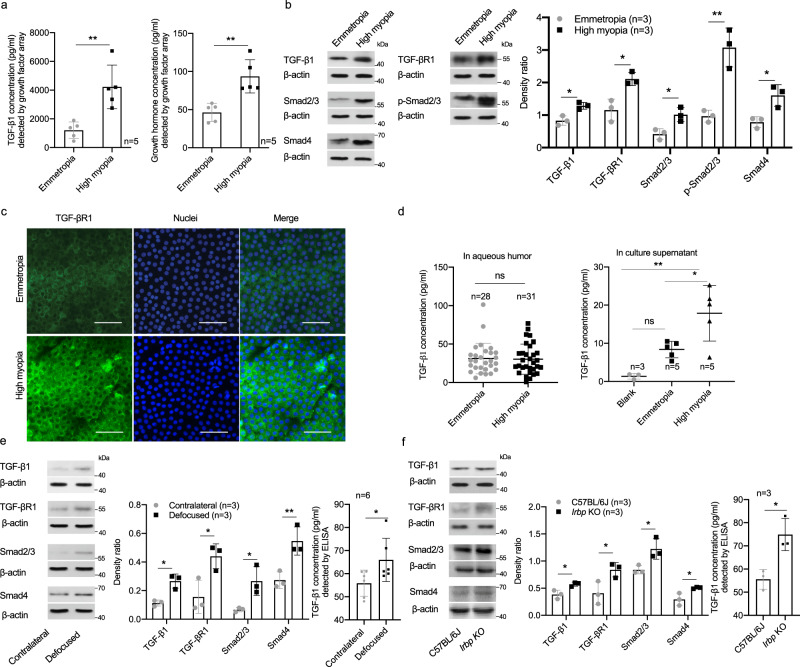
Aberrant activation of TGF-β1-Smads signaling in lens of high myopia. **a** Detection of TGF-β1 and growth hormone levels in human lens epithelium by growth factor array (*n* = 5, both *p* = 0.003). **b** Examination of protein expression by Western blotting of TGF-β1, TGF-βR1, and downstream Smad2/3, p-Smad2/3, and Smad4 (*n* = 3, *p* = 0.012, *p* = 0.013, *p* = 0.019, *p* = 0.004, and *p* = 0.019, respectively). Right, the band density in Western blotting was normalized to loading control as a ratio for statistical analysis. **c** Immunofluorescence images of TGF-βR1 staining in human lens epithelium (*n* = 5). Scale bar: 50 μm. **d** Measurement of human TGF-β1 concentration with ELISA in aqueous humor (*n* = 28 in the control group vs. *n* = 31 in the highly myopic group, *p* = 0.876) and primary lens epithelial cell culture supernatant (3, 5, and 5 different cultures in the blank, emmetropic control, and highly myopic group, respectively, one piece of lens epithelium in one culture for this assay, bland vs. emmetropia *p* = 0.164, emmetropia vs. high myopia *p* = 0.027, blank vs. high myopia *p* = 0.002). Blank refers to DMEM without culture of lens epithelium. **e** Western blotting of protein expression of TGF-β1, TGF-βR1, Smad2/3, and Smad4 (*n* = 3, *p* = 0.012, *p* = 0.041, *p* = 0.048 and *p* = 0.008, respectively) and ELISA test of TGF-β1 level in mouse lens epithelium of defocus-induced highly myopic eye and the contralateral eye (*n* = 6, *p* = 0.016). Middle, the band density in Western blotting was normalized to loading control as a ratio for statistical analysis. **f** Western blotting of protein expression of TGF-β1, TGF-βR1, Smad2/3, and Smad4 (*n* = 3, *p* = 0.021, *p* = 0.040, *p* = 0.032, and *p* = 0.033, respectively) and ELISA test of TGF-β1 level in lens epithelium of *Irbp* KO mice and wild type C57BL/6J (*n* = 3, *p* = 0.015). Middle, the band density in Western blotting was normalized to loading control as a ratio for statistical analysis. *n* = biological replicates. In (**a**, **b**, **e**, and **f**), pooled samples were used. Results are expressed as mean ± SD. Level of significance was detected using two-sided Student’s *t* test (Figs. a, b, and f) and two-sided paired *t* test (Fig. e). In (**d**), TGF-β1 concentration in aqueous humor was analyzed using two-sided Student’s *t* test, while the concentration in culture supernatant was analyzed using one-way ANOVA with Turkey’s multiple comparisons test for further comparison between two groups. **p < 0.01, *p < 0.05 and ns represents no significant difference. Source data are provided as a Source Data file.

To further identify the factors that could have contributed to aberrant lens growth in high myopia, we conducted a protein microarray of 40 growth factors using pooled human lens epithelial samples. Higher TGF-β1 (4226.72 ± 1, 516.86 vs. 1, 212.46 ± 572.18 pg/ml in control, *p* = 0.003) and growth hormone (93.64 ± 21.85 vs. 46.3 ± 11.87 pg/ml in control, *p* = 0.003) in lens epithelium of highly myopic eyes were identified (each sample = a pool of four lens epithelial samples, n= biological replicates, Fig. 6a) and the elevated TGF-β1 was verified by Western blotting (Fig. 6b). Western blotting and immunofluorescence staining showed up-regulation of TGF-βR1, as well as the key signaling molecules downstream of TGF-βR1 activation (Smad2/3, p-Smad2/3, and Smad4) (Fig. 6b, c).

ELISA assay of aqueous humor showed no difference in TGF-β1 concentration between highly myopic and emmetropic eyes (Fig. 6d). In contrast, significantly higher TGF-β1 was found in the supernatant in primary culture of LECs from highly myopic subjects (approximately 1.1 × 10^5^ cells per culture, Fig. 6d), suggesting that TGF-β1 could affect LECs in an autocrine manner.

Higher protein level of TGF-β1 as well as key signaling molecules including TGF-βR1, Smad2/3, and Smad4 was also identified in the lens of two mouse models of high myopia using pooled mouse lens epithelial samples by Western blotting and the level of TGF-β1 was further verified by ELISA (Fig. 6e, f).

### Activated TGF-β1-Smad signaling promotes β/γ-crystallin expression and LEC proliferation

**Fig. 7:**
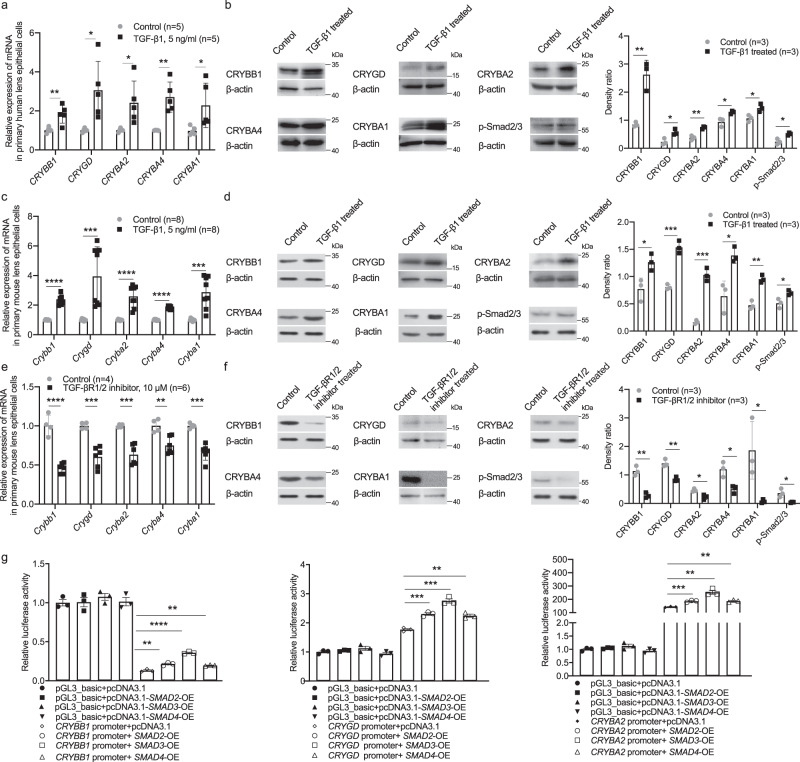
Up-regulation of β/γ-crystallins by TGF-β1. **a**, **b** Changes of β/γ-crystallin gene expression detected by qPCR (*n* = 5, *CRYBB1 p* = 0.005, *CRYGD p* = 0.015, *CRYBA2 p* = 0.022, *CRYBA4 p* = 0.001, *CRYBA1 p* = 0.036) and Western blotting (*n* = 3, CRYBB1 *p* = 0.004, CRYGD *p* = 0.021, CRYBA2 *p* = 0.003, CRYBA4 *p* = 0.022, CRYBA1 *p* = 0.027) and changes of p-Smad2/3 level detected by Western blotting (*n* = 3, *p* = 0.013) in primary human LECs from emmetropic eyes after TGF-β1 treatment (5 ng/ml for 24 h). Right, the band density in Western blotting was normalized to loading control as a ratio for statistical analysis. **c**, **d** Changes of β/γ-crystallin gene expression detected by qPCR (*n* = 8, *Crybb1 p* = 4.65e−8, *Crygd p* = 0.0009, *Cryba2 p* = 2.53e-5, *Cryba4 p* = 3.58e-11, *Cryba1 p* = 0.0002) and Western blotting (*n* = 3, CRYBB1 *p* = 0.029, CRYGD *p* = 0.0005, CRYBA2 *p* = 0.0003, CRYBA4 *p* = 0.015, CRYBA1 *p* = 0.001) and changes of p-Smad2/3 level detected by Western blotting (*n* = 3, *p* = 0.027) in primary cultured mouse LECs with TGF-β1 treatment (5 ng/ml for 24 h). Right, the band density in Western blotting was normalized to loading control as a ratio for statistical analysis. **e**, **f** Changes of β/γ-crystallin gene expression detected by qPCR (*n* = 4 in control and 6 in the treated group, *Crybb1 p* = 2.05e-5, *Crygd p* = 0.0004, *Cryba2 p* = 0.0005, *Cryba4 p* = 0.004, *Cryba1 p* = 0.0003) and Western blotting (*n* = 3, CRYBB1 *p* = 0.002, CRYGD *p* = 0.003, CRYBA2 *p* = 0.015, CRYBA4 *p* = 0.016, CRYBA1 *p* = 0.038) and changes of p-Smad2/3 level detected by Western blotting (*n* = 3, *p* = 0.021) in primary cultured mouse LECs with TGF-βR1/2 inhibitor treatment (LY2109761, 10 μM for 24 h). Right, the band density in Western blotting was normalized to loading control as a ratio for statistical analysis. **g** Elevated transcriptional activity of crystallin genes with co-transfection of *SMAD*s plasmid (*n* = 3; *CRYBB1* promoter: *SMAD2 p* = 0.004, *SMAD3 p* = 6.36e-5, *SMAD4 p* = 0.004; *CRYGD* promoter: *SMAD2 p* = 0.0007, *SMAD3 p* = 0.0003, *SMAD4 p* = 0.002; *CRYBA2* promoter: *SMAD2 p* = 0.0008, *SMAD3 p* = 0.002, *SMAD4 p* = 0.001). *n*=biological replicates. Results are expressed as mean ± SD. Level of significance was detected by two-sided Student’s *t* test (**a**–**g**). ****p < 0.0001, ***p < 0.001, **p < 0.01 and *p < 0.05. Source data are provided as a Source Data file.

We treated LECs with TGF-β1 of different concentration gradients and detected a stimulatory effect on cell growth at 5 ng/ml. At 5 ng/ml, TGF-β1 treatment significantly increased the mRNA and protein levels of *CRYBB1, CRYGD, CRYBA2, CRYBA4,* and *CRYBA1* in primary LECs collected from human emmetropic eyes (Fig. 7a, b) and activated the downstream signaling pathway as shown by the elevated level of p-Smad2/3 (Fig. 7b).

In primary mouse LECs, TGF-β1 (5 ng/ml) also increased the expression of *Crybb1*, *Crygd, Cryba2, Cryba4,* and *Cryba1* and activated the ensuing pathway manifest as up-regulated p-Smad2/3 just as the results in human primary LECs (Fig. 7c, d); in contrast, the selective TGF-βR1/2 inhibitor LY2109761 induced the opposite effects (Fig. 7e, f).

Dual-luciferase reporter assay showed significantly elevated transcriptional activity of *CRYBB1*, *CRYGD* and *CRYBA2* in 293T cells with plasmid co-transfection of effector molecules of TGF-β1 signaling pathway: *SMAD2*, *SMAD3* and *SMAD4* (Fig. 7g).

**Fig. 8:**
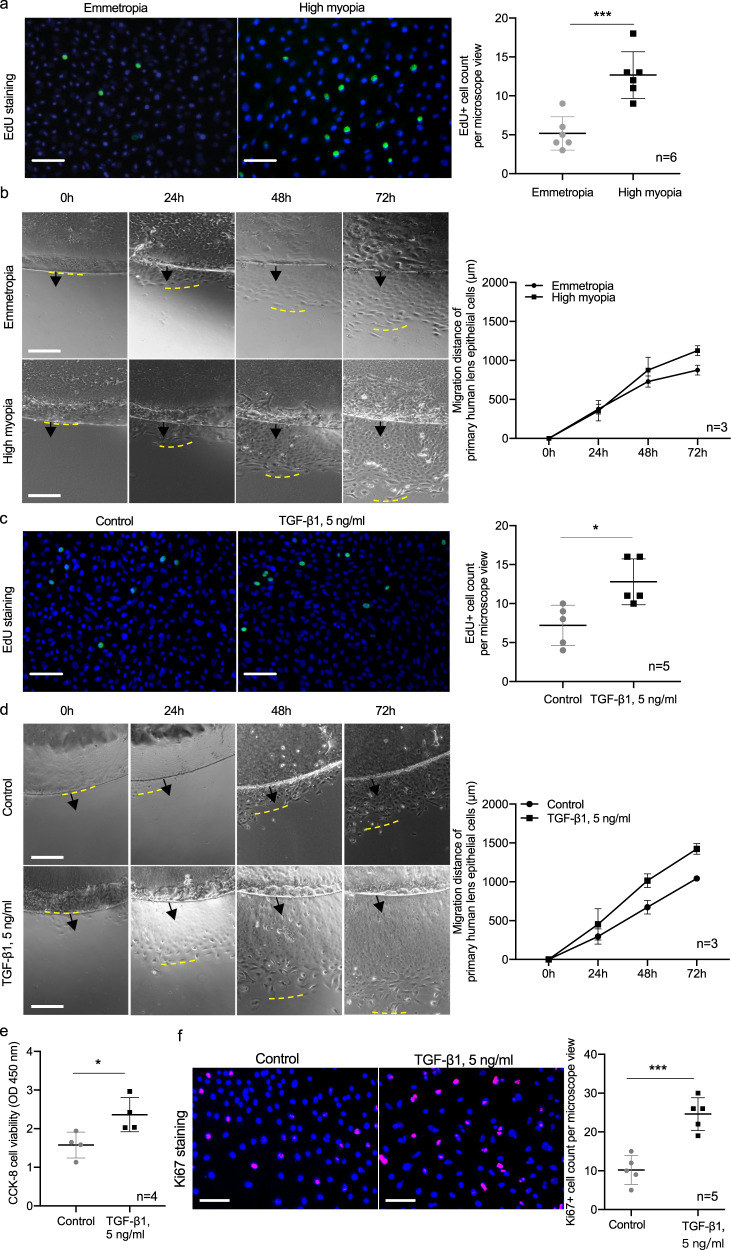
Promoted proliferation of lens epithelial cells by TGF-β1. **a** EdU incorporation assay of primary human LECs from emmetropic and highly myopic eyes (*n* = 6, *p* = 0.0006). Scale bar: 100 μm. **b** Primary culture of human LECs showing the migration of LECs from the rim of epithelium (*n* = 3, *p* = 0.041). Scale bar: 500 μm. Black arrows show the original lens epithelium rim and the yellow dashed lines refer to the place where proliferating LECs outreached. **c** EdU incorporation assay of primary human LECs from emmetropic eyes after TGF-β1 treatment (5 ng/ml for 24 h, *n* = 5, *p* = 0.013). Scale bar: 100 μm. **d** Primary culture of human LECs from emmetropic eyes showing the migration of LECs from the edge of epithelium after TGF-β1 treatment (5 ng/ml for 24 h, *n* = 3, *p* = 0.002). Scale bar: 500 μm. Black arrows show the original lens epithelium rim and the yellow dashed lines refer to the place where proliferating LECs outreached. **e** CCK-8 cell viability assay of primary human LECs with and without TGF-β1 treatment (5 ng/ml for 24 h, *n* = 4, *p* = 0.030). **f** Ki67 staining of primary human LECs with and without TGF-β1 treatment (5 ng/ml for 24 h, *n* = 5, *p* = 0.0004). Scale bar: 100 μm. *n* = biological replicates. Results are expressed as mean ± SD. Level of significance was detected by two-sided Student’s *t* test (**a**, **c**, **e**, and **f**) and repeated measures ANOVA (**b** and **d**), multiple comparison was not conducted). ***p < 0.001 and *p < 0.05. Source data are provided as a Source Data file.

In addition, primary LECs collected from highly myopic subjects exhibited faster proliferation than those from emmetropic subjects (Fig. 8a, b) and a similar effect was achieved in primary LECs from emmetropic subjects with TGF-β1 treatment (Fig. 8c–f).

### Activation of TGF-β1-Smad signaling by MAF up-regulated β/γ-crystallin expression in high myopia conditions

We further investigated a possible crosstalk between MAF and TGF-β1-Smad signaling in regulating lens β/γ-crystallin expression.

**Fig. 9:**
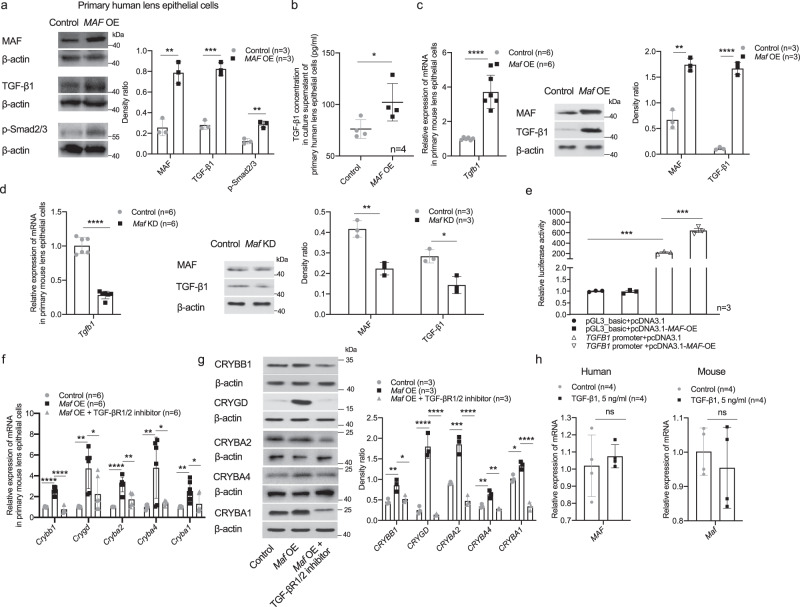
Activation of TGF-β1-Smad signaling by MAF up-regulated β/γ-crystallin expression. **a** Elevated expression of TGF-β1 and p-Smad2/3 in primary human LECs with *MAF* overexpression (*n* = 3, MAF *p* = 0.002, TGF-β1 *p* = 0.0002, p-Smad2/3 *p* = 0.002). Right, the band density in Western blotting was normalized to loading control as a ratio for statistical analysis. **b** Elevated secretion of TGF-β1 into cell culture media of primary human LECs with *MAF* overexpression (*n* = 4, *p* = 0.043). **c**, **d** Changes of *Tgfb1* expression in primary mouse LECs in accordance with *Maf* changes as detected by qPCR (both *n* = 6, *p* = 4.36e−5 and *p* = 1.16e−7) and Western blotting (*n* = 3, *Maf* OE: MAF *p* = 0.001, TGF-β1 *p* = 2.30e-5; *Maf* KD: MAF *p* = 0.003, TGF-β1 *p* = 0.010). Right, the band density in Western blotting was normalized to loading control as a ratio for statistical analysis. **e** Elevated transcriptional activity of *TGFB1* gene with co-transfection of *MAF* plasmid (*p* = 0.0007). **f**, **g** Changes of β/γ-crystallin gene expression detected by qPCR (*n* = 6, *Crybb1*: control vs. *Maf* OE and *Maf* OE vs. *Maf* OE + TGF-βR1/2 inhibitor *p* = 3.30e−7 and *p* = 6.23e−8; *Crygd*: *p* = 0.001 and *p* = 0.027, *Cryba2*: *p* = 1.7e−5 and *p* = 0.001; *Cryba4*: *p* = 0.005 and *p* = 0.011; *Cryba1*: *p* = 0.007 and *p* = 0.028) and Western blotting (*n* = 3, CRYBB1: *p* = 0.008 and *p* = 0.019, CRYGD: *p* = 9.2e−5 and *p* = 6.2e−5, CRYBA2: *p* = 0.0005 and *p* = 6.4e−5, CRYBA4: *p* = 0.003 and *p* = 0.001, CRYBA1: *p* = 0.015 and *p* = 3.5e−5) with *Maf* overexpression ± TGF-βR1/2 inhibitor treatment (10 μM for 24 h) in primary mouse LECs. Right, the band density in Western blotting was normalized to loading control as a ratio for statistical analysis. **h** No significant change of MAF detected by qPCR (both *n* = 4, *p* = 0.586 and *p* = 0.511) in primary human or mouse LECs after TGF-β1 treatment (5 ng/ml, 24 h). *n* = biological replicates. Results are expressed as mean ± SD. Level of significance was detected using two-sided Student’s *t* test (**a**–**e**, and **h**) and one-way ANOVA plus Tukey’s multiple comparisons test (**f** and **g**). ****p < 0.0001, ***p < 0.001, **p < 0.01, *p < 0.05 and ns represents no significant difference. Source data are provided as a Source Data file.

In primary human LECs, *MAF* overexpression elevated the expression of TGF-β1 and p-Smad2/3 at the same time (Fig. 9a) and the secretion of TGF-β1 into cell culture media was also enhanced (Fig. 9b). In primary mouse LECs, *Maf* overexpression increased the expression of TGF-β1 (Fig. 9c); *Maf* knockdown, in contrast, inhibited TGF-β1 expression (Fig. 9d). Dual-luciferase reporter assay showed significantly elevated transcriptional activity of *TGFB1* gene with co-transfection of *MAF* plasmid in 293T cells (Fig. 9e). In contrast, the TGF-βR1/2 inhibitor LY2109761 attenuated the up-regulation of β/γ-crystallins induced by *Maf* overexpression in primary mouse LECs (Fig. 9f, g), suggesting that TGF-β1-Smad pathway might be one downstream target of MAF, which in turn regulated the expression of β/γ-crystallin genes.

Conversely, we examined the possible regulation of MAF by TGF-β1 both in primary human and mouse LECs. TGF-β1 treatment (5 ng/ml) did not elevate the expression of MAF in either primary human or mouse LECs (Fig. 9h).

## Discussion

High myopia is a blinding ocular disease with increasing prevalence worldwide. Its pandemic trends are compelling clinicians and researchers to better understand the disease. Though a great deal of studies have tried to unveil the pathogenesis of onset and progression of myopia, how crystalline lens, the core refracting medium of the eye, changes in this process remains poorly understood. Meanwhile, complications of lens replacement surgery such as intraocular lens malposition frequently occurred, suggesting a pathological change of lens size in highly myopic eyes. Our study compared the dimensions of the lens between highly myopic and emmetropic eyes in large samples using MRI and demonstrated the increased equatorial diameter and the flattened anterior surface of the lens in high myopia, which morphologically addressed the unanswered question. Furthermore, increased expressions of genes encoding β/γ-crystallins were identified in the lens epithelium of highly myopic subjects, which were also positively correlated with the lens size. The bigger size and increased β/γ-crystallins were also observed in the lens of two independent mouse models of high myopia. Further explorations unveiled the increased MAF in high myopia and its participation in a direct activation of crystallin promoters and an indirect activation via TGF-β1-Smad signaling. Together, our study identified a previously unrecognized role for MAF-TGF-β1-crystallin axis in pathological growth of lens in high myopia.

Pathological growth of lens in human highly myopic eyes is mainly reflected on the larger equatorial diameter and flatter anterior surface. Previously, only lens thickness has been studied due to the limitation of ophthalmic instruments. Hashemi et al. found that lens thickness in highly myopic eyes was not larger than that in the emmetropic group^21^. Franco et al followed ocular parameter variations of a pediatric population with duration of nine months and found that in spite of the significant elongation of the eyeball, there was no significant variation of lens thickness in these children^22^. As to the lens equatorial diameter, very few studies have addressed this problem. In our study, using ocular MRI data acquired from digital picture archiving system of our hospital, we found significantly larger lens equatorial diameters in highly myopic eyes, which helps explain the reason why in these eyes, postoperative intraocular lens malposition was frequently seen and impaired the visual outcomes^10,11^, which is of great clinical relevance. Our findings thus call attention to appropriate IOL selection for highly myopic eyes in clinical practice. Larger equatorial diameter and flatter anterior surface of myopic lens might also induces myopia progression through increased peripheral hyperopic defocus during childhood^23–26^. Yet future studies are warranted to verify this assumption.

The morphological changes of lens may be influenced by changes in crystallins. In highly myopic subjects, expressions of five β/γ-crystallins (together make up of 28.0% of the total protein of normal lens, Supplementary Fig 3) were significantly up-regulated in their lens epithelium and showed a positive correlation with the lens size, and similar findings were also observed in two independent mouse models of high myopia. One may also notice that the crystallin data in the lens mass were not exactly the same as those observed in the lens epithelium. To interpret the differences, it is important to bear in mind that they are formed at different stages of the lens life. The composition of lens mass is mainly from prenatal embryonic and fetal nucleus and a small portion of adult nucleus. Thus, it is the lens epithelium that is the true source for the generation of adult lens fibers, as well as the main contributor of difference in lens diameters between highly myopic and emmetropic eyes in adults. Hence, our study mainly focused on and displayed data using lens epithelium, which archived informative and consistent results amid various assays in both human and mouse samples.

MAF, a transcription factor previously reported to control embryonic lens development, was found to be significantly up-regulated and participate in the direct activation of crystallin expression in postnatal lens of highly myopic eyes^27,28^. In fetal mice, MAF is expressed in the lens vesicle after invagination and becomes up-regulated in the equatorial zone of the lens. Besides, several mutations in MAF have been associated with distinct forms of congenital cataract resulting from reduced expression of β/γ-crystallins^29,30^. However, the data scarcity in postnatal regulation of crystallins makes it difficult to understand the role of MAF in grownups. In our study, we found with surprise that in the lens of adults, MAF remained significantly activated in highly myopic eyes compared with emmetropic eyes, and its direct recruitment to promoters of β/γ-crystallin genes was also elevated, together contributing to increased β/γ-crystallins in these eyes. In combination with the significant association between increased β/γ-crystallins and a larger lens size, these findings reveal that the enlargement of the lens in highly myopic eyes might be associated with the up-regulated β/γ-crystallins induced by activated MAF after birth.

As to the pathological growth of lens in high myopia, apart from the direct regulation of crystallins by MAF, it is also tempting to speculate on the participation of growth factors in this process^31–33^. Thus, we profiled 40 common growth factors in lens epithelium from highly myopic subjects, and were surprised to identify the significantly elevated level of TGF-β1. What’s more, the expression of TGF-β1 receptor and effector molecules such as Smad2/3, p-Smad2/3 (the activated form of Smad2/3) and Smad4 (the co-worker of p-Smad2/3 in transcription activation of target genes) were all elevated in the lens of human and mouse highly myopic eyes, further confirming the enhanced signaling of TGF-β1-Smad in high myopia conditions.

Besides, as revealed in our results, the aberrant activation of TGF-β1-Smad signaling in the lens of highly myopic eyes was in an autocrine manner. Patrick et al. reported that autocrine TGF-β1 signaling could be enhanced in response to activation of a receptor tyrosine kinase in hepatocellular carcinoma epithelial cells^34^. Christina et al. observed the induction of TGF-β signaling by the down-regulation of endogenous inhibitors of autocrine signals in mammary epithelial cells^35^. In this study, TGF-β1 production inside LECs could be secreted and the TGF-β1 secretion of primary LECs from highly myopic eyes was greater than that of the emmetropic eyes.

With regards to the function of TGF-β1 signaling, previous studies have reported a variety of cellular responses depending on the cellular context, such as proliferation, migration, apoptosis, extracellular matrix formation, etc.^36–39^. As found in our study, in LECs, stimulation by low-concentration of TGF-β1 significantly increased the expressions of β/γ-crystallins via activation of ensuing Smad pathway. In contrast, inhibition of this pathway using inhibitor of TGF-β1 receptor induced the opposite results. In addition, TGF-β1 stimulation also enhanced the proliferation of primary LECs. Calvier et al. reported the promotion of proliferation of vascular smooth muscle cells by TGF-β1^40^ and Lee et al. found that TGF-β1 could promote cell cycle progression of retinal pigment epithelial cells^37^. Together, TGF-β1-Smad signaling promotes the crystallin expression and proliferation of LECs in highly myopic eyes.

**Fig. 10:**
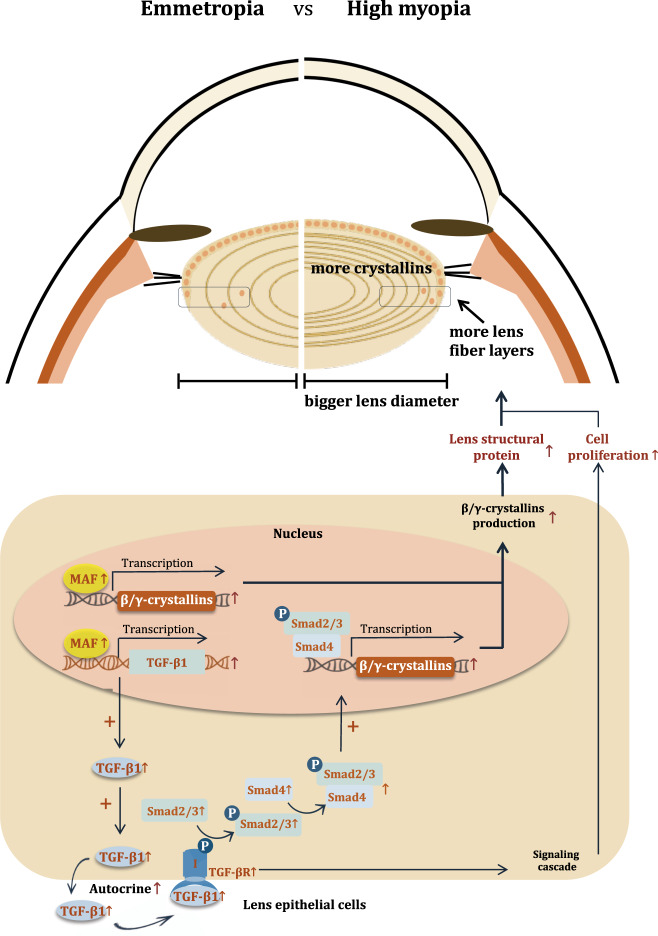
Schematic illustration of the MAF-TGF-β1-crystallin axis that underlies the pathological growth of lens in high myopia. Elevated MAF in lens of highly myopic eyes could promote β/γ-crystallin expressions by direct binding to their promoters and by boosting autocrine secretion of TGF-β1 and subsequent Smad signaling pathway, together contributing to larger equatorial diameter of lens in highly myopic eyes.

In high myopia, the aberrant activation of TGF-β1 signaling was attributed to the positive regulation by MAF. After the discovery of elevated MAF and TGF-β1 signaling, we sought to find the possible crosstalk between them in regulating the cellular process inside LECs. TGF-β1 treatment failed to increase *MAF* expression in primary LECs; however, the overexpressed MAF could elevate the TGF-β1 secretion and activate the subsequent pathway with elevated p-Smad2/3. Furthermore, luciferase reporter assay confirmed the positive activation of *TGFB1* gene transcription by MAF, which provides the explanation for the partially attenuated up-regulation of crystallins in primary LECs with overexpressed MAF by interference with TGF-βR1/2 inhibitor, indicating that TGF-β1 signaling was a downstream pathway of MAF mediating an indirect up-regulation of crystallins. Together with elevated MAF and TGF-β1 signaling transducers found in the lens of highly myopic eye, it could be speculated that MAF might up-regulate crystallin expression in lens of high myopia by a direct activation of their promoters and by simultaneous activation of downstream TGF-β1-Smad signaling, which together underlies the pathological growth of lens in these eyes (Fig. 10). The MAF- TGF-β1-crystallin axis identified in this study may be also highly suggestive of other lens diseases. The persistent high expression level of lens crystallins might also contribute to the earlier formation of nuclear cataract in highly myopic eyes possibly due to the over-compaction of lens tissue. If such association can be established, it will provide novel therapeutic targets for prevention and treatment of cataract in highly myopic eyes in the future. Still, further studies are warranted.

Of note, as many researchers have suggested, immortalized LEC lines lack appropriate expression of crystallin genes thus might not be an ideal material to study the regulation of crystallins^41,42^. Thus, in our study, we used the primary LECs from human and mouse with stable expression of crystallin genes and treated them for only 24 h, which improve the accuracy and stability of the results^43^.

To conclude, our study demonstrates morphological changes of lens as a characteristic feature of high myopia, and point to the dysregulation of a MAF-TGF-β1-crystallin axis as a novel molecular mechanism underlying the pathological lens growth, which could provide a potential guide for future therapeutic interventions.

## Methods

This study was affiliated with the Shanghai High Myopia Study and was reviewed and approved by the Ethics Committee of the Eye & Ear, Nose, and Throat (ENT) Hospital of Fudan University, Shanghai, China in accordance with applicable regulations. Written informed consents were obtained before surgery from each and all patients for the use of their clinical data and biosamples including lens epithelium, lens mass, and aqueous humor. All procedures adhered to the tenets of Declaration of Helsinki. Animal experiments were approved by the Ethics Committee for Animal Studies of Eye & ENT Hospital of Fudan University and experimental procedures all conformed to the ARVO Statement for the use of animals in research. Our study does not involve export or publication of any genetic resources at individual levels therefore the Guidance of the Ministriy of Science and Technology for the Review and Approval of Human Genetics Resources do not apply to our study.

### Patients

In this study, high myopia was defined as eyes with an axial length of ≥26.00 mm, and emmetropia was defined as eyes with an axial length between 22.00 and 24.50 mm.

#### Patients for MRI data acquisition

To compare the lens dimensions between highly myopic and emmetropic patients, MRI data of a total of 249 eyes of 249 patients were retrieved from the digital picture archiving and communication system of our hospital between March 2017 and June 2019. Here, G*Power Software 3.1 was used for sample size calculation with a desired significance level of 5% and test power of 0.95. Reasons for referral included the diagnostic workup of various ocular and orbital mass lesions as well as trauma in one eye and only the healthy contralateral eyes with confirmations from both experienced eye doctors and radiologists were included in this study. To conduct the 3D reconstruction of lens volume, five highly myopic subjects and five emmetropic subjects received ocular MRI with a thin slice thickness cut method. To examine the correlation between expression levels of target genes and the lens size, another 48 highly myopic lens surgery candidates were recruited for preoperative ocular MRI and lens epithelial tissues were collected intraoperatively.

#### Patients and tissue sample collection

Patients with complaints of decreased visual function who seeked for lens replacement surgery at Eye & ENT hospital were screened. The highly myopic group was recruited according to the following criteria: (1) patients who wanted correction of high myopia through lens replacement, or (2) bilateral highly myopic patients who needed refractive lens surgery in “a relatively normal second eye” to correct the severe anisometropia after the first-eye lens surgery. The emmetropic group was recruited as the following criteria: patients who required the correction of presbyopia through lens replacement surgery due to decreased accommodation and difficulties in focusing on near objects and fine prints. Lenses of these patients were close to or had slightly increased density than those of normal population of the same age. Exclusion criteria were ocular comorbidities such as glaucoma and uveitis, history of ocular surgery or trama, or systemic diseases such as diabetes and malnutrition.

#### Ocular sample acquirement and preservation

Lens epithelium, lens mass and aqueous humor specimens were collected during standard lens replacement surgery^5^. To clarify at first, only one piece of lens epithelium (one specimen), one lens mass and 100–200 μl of aqueous humor could be acquired from one eye during a lens surgery. Aqueous humor was obtained at the beginning of the surgery: before making the incision, anterior chamber paracentesis was performed with a 1 ml graduated syringe and aqueous humor was then aspirated without touching peripheral tissues. By the subsequent standard step of capsulorhexis during lens surgery, the lens epithelium was peeled off and collected (lens epithelium would otherwise be discarded if not for research purpose). Lens mass was acquired during the lens surgery using a traditional technique of extracapsular lens extraction. Based on the experimental requirement, totally 463 pieces of lens epithelia, 18 lens masses, 59 specimens of aqueous humor (sample for highly myopic/ emmetropic groups were: 240/ 223, 8/ 10, and 31/ 28, respectively) were collected from 248 highly myopic patients and 233 emmetropic patients, respectively (specimens of only one eye from one patient were collected, and different kinds of specimens could be either collected from one patient or from different ones). Samples were immediately stored at −80 °C until further analysis. For immunofluorescence staining within 24 h or primary culture, samples were kept in Dulbecco’s Modified Eagle Medium (DMEM, Sigma-Aldrich, USA) supplemented with 20% fetal bovine serum (FBS, #10099141, Gibco, USA).

#### Sample usage

With regard to the usage of the specimens, for assays including gene expression microarray and immunofluorescence staining, lens epithelium specimen from an individual was used as one sample. For qPCR, Western blotting, PRM, ChIP-qPCR, and human growth factor array, 3-6 pieces of lens epithelial samples were pooled as one due to the limited amount of RNA or proteins that can be extracted from one piece. In primary human LEC culture, three pieces of human lens epithelial samples were placed in one culture dish, except that in the examination of TGF-β1 autocrine secretion, only one piece of lens epithelium was used in one culture. Lens mass and aqueous humor obtained from an individual were used as one sample.

### Two mouse models of high myopia

Mice were bred and housed in clear cages and maintained at a temperature of 21 °C with 40–60% humidity with a 12:12-h light-dark cycle (light on at 7:00 AM and off at 7:00 PM).

#### Defocus-induced Highly Myopic Mouse

Four-week-old male C57BL/6J mice obtained from SLAC Laboratory Animal Co. Ltd. (China) were used to make the defocus-induced high myopia model by wearing a −25.00D lens onto the peri-orbital skin of the right eye and the left eye served as the control. An infrared photorefractor (Steinbeis Transfer Center, Germany) was used to measure the refractive state at the beginning of the study and only mice with less than 1.00D difference of refraction between the two eyes could be used. Mice were checked every day to make sure the attachment of the lens. After 4 weeks, refraction of mice was measured again. Only mice with the right eye showing at least 6.00D of myopic shift compared with the left eye were taken as a successful model of defocus-induced high myopia.

#### Irbp KO spontaneous highly myopic mice

*Irbp* KO mice (#023080) were purchased from the Jackson Laboratory (https://www.jax.org/jax-mice-and-services). The *Irbp* KO mouse may undergo rapid eye size growth than the wild type C57BL/6J mice and the retina of *Irbp* KO mice manifests similar to the retina of the highly myopic eye of human^44^. Thus, this type of mice was also used in our study to investigate the lens changes of highly myopic eyes in mice. Refraction was measured at eight weeks after birth in comparison with the C57BL/6J mice of the same age.

#### Sample acquirement and preservation

Mice were euthanized by cervical dislocation and eyeballs were enucleated. To make a paraffin section for further staining, eyeballs were fixed in 4% paraformaldehyde. To obtain the lens, dissections were immediately conducted after the enucleation of the eyeballs. Placed in PBS, adherent tissue such as iris and retina were carefully removed by fine forceps and ophthalmic scissors. Lens epithelium was peeled off by forceps and immediately stored at −80 °C until further experiments. Samples for primary culture were treated similarly to that of human samples.

#### Sample usage

In gene expression profiling, qPCR, Western blotting, PRM, and primary mouse LEC culture, 3–6 pieces of mouse lens epithelial samples were pooled as one due to the limited amount of RNA or proteins within one piece. In the ELISA to measure the TGF-β1 level in mouse lens epithelium, two pieces were pooled and used as one sample.

### MRI

#### Human ocular MRI

Human ocular MRI was conducted using a 3.0 Tesla scanner using a 32-channel head coil (Verio; Siemens, Erlangen, Germany), with the following parameters: transverse T2-weighted turbo spin echo, TR 4390 ms, TE 96 ms, slice thickness 3 mm, slice gap 0.3 mm, field of view 180*180 mm, average 2, and voxel size = 0.3 × 0.3 × 3.0 mm. The axial length, the equatorial diameter, the thickness, and the maximum cross-sectional area of the lens were measured on the post-processing workstation provided by the manufacturer (Siemens, Erlangen, Germany). Lens volume was calculated based on 3D reconstruction from T2-weighted MRI with a thin slice thickness cut method using an open-source software (3D Slicer 4.10.1 r27931, https://www.slicer.org/, examples were shown in Supplementary Fig 4).

#### Mouse ocular MRI

High-resolution 7.0 Tesla MRI (Biospec 70/20 USR, Bruker) was performed for imaging eyeballs of the mouse^45^. The axial length of eyeball and the maximum cross-sectional area of lens were measured with ParaVision® 6.0.1 software (Bruker, USA).

### Primary LEC culture

In primary human LEC culture, human lens epithelial samples were placed in culture dish with LECs facing upward and incubated in DMEM supplemented with 20% FBS and were grown at 37 °C and 5% CO_2_ with medium changed every other day.

Primary mouse LEC culture used lens epithelial samples that were dissected from 3-week C57BL/6J mice. The culture method was the same as that of primary human LEC.

### Gene expression profiling and Ingenuity pathway analysis

RNA was isolated from tissues or cells using the RNeasy Micro Kit (#74004, Qiagen, Germany). Comparative microarray analysis of human lens epithelium was performed using HG-U133 Plus 2.0 Array (Affymetrix, USA). Differential gene expression was determined using the *limma* statistical package (3.26.9) as described at http://www.bioconductor.org. Differentially expressed genes (DEGs) are defined by fold change of >2.0 plus *p* < 0.05. The Ingenuity Pathway Analysis was used to identify common molecules that regulate the up-regulated lens structural protein encoding genes, conducted to establish a self-defined network targeting selected differentially expressed genes (*CRYBB1*, *CRYGD*, *CRYBA2*, *CRYBA4*, *LGSN*, *LIM2*, *CRYBA1,* and *BFSP1*) that were associated with lens.

Comparative microarray analyses of lens epithelium from two mouse models of high myopia were also conducted. Agilent SurePrint G3 Mouse Gene Expression Microarray was used and also performed by Shanghai Biotechnology Corporation.

### ChIP-qPCR

Formaldehyde cross-linked chromatin was firstly obtained from human lens epithelium. The sheared chromatin (200–1000 bp) was then generated by sonication. Chromatin acquired from lens epithelium of high myopes and emmetropes was incubated with anti-MAF antibody (Abcam, UK). The immunoprecipitates were washed and resuspended in the buffer. Genomic DNA was eluted using QIAquick Spin Gel Purification Kit (Qiagen). The eluted DNA was reverse cross-linked at 65°C overnight and used for the qPCR using SYBR Green qPCR mix which could determine the amount of DNA fragment representing promoters of *CRYBB1*, *CRYGD*, *CRYBA2*, *CRYBA4*, and *CRYBA1*. Prediction of MAF binding sites in promoters of crystallin genes were conducted using the online Jaspar database (http://jaspar.genereg.net/). The sequences of primers used in qPCR part were listed in Supplementary Table S1.

### qPCR, Western blotting, and Immunofluorescence staining

Total RNA from lens epithelium was extracted using the RNeasy Micro Kit (#74004, Qiagen, Germany). RNA quantitation was performed using a Nanodrop spectrophotometer (Thermo Fisher Scientific, USA) and RNA was reverse transcribed into cDNA using the Primescript RT reagent kit (#RR047, Takara, Japan). mRNA levels of selected genes were quantified by SYBR Green-based real-time PCR on an ABI 7500 Analyzer (Thermo Fisher Scientific).

For Western blotting, protein extracts obtained using RIPA lysis buffer were separated by SDS-PAGE and electrotransferred onto PVDF membrane followed by blocking and the exposure to primary and secondary antibodies. Proteins were finally visualized by using Pierce Western Blotting Substrate Plus (Thermo Fisher Scientific, USA). Band densities were assessed and normalized to loading control (β-actin) as a ratio for further statistical analyses.

In immunofluorescence staining, lens epithelium was attached to the slide with LEC side facing upward and fixed in 4% paraformaldehyde for 30 min. Epithelia were then permeabilized with PBS containing 0.3% Triton X-100 and then blocked and probed with primary antibodies at 4 °C overnight. Secondary antibodies and Hoechst were used to visualize the stained cells. Slides were observed under a confocal microscope (Leica Microsystems, Germany).

The assay was at least repeated three times. Primer sequences and antibodies (with dilution information) are listed in Supplementary Table S2 and S3.

### Parallel reaction monitoring

PRM analysis, a mass spectrometry technique was used to assess the relative abundance of crystallins in lens epithelium and lens mass. One unique peptide was selected for quantification of each targeted protein (peptide sequences seen in Supplementary Table S4).

### Shot-gun proteomic analysis

To analyze the percentage contribution of crystallins in human lens, shot-gun proteomic analysis was conducted. For crystallins of interest, we calculated their percentage contribution to total protein by dividing the iBAQ value for each protein by sum of iBAQ values across all proteins.

### Growth factor screening by a human antibody array

A biotin label-based human antibody array including 40 growth factors (QAH-GF-1, RayBiotech, USA) was used according to the manufacturer’s protocol. After determination of concentrations of total protein extracted from human lens epithelial samples, 50 μg protein of each sample was diluted into 100 μl and then hybridized to the arrays.

### ELISA

Human TGF-β1 ELISA Kit (#EK0513, Boster Biological Technology, China) was used for the quantification of TGF-β1 concentration in aqueous humor and the supernatant of cultured primary human LECs to examine the autocrine production of TGF-β1 by LECs. Quantikine ELISA Mouse TGF-β1 Immunoassay (#MB100B) was used for the quantitative determination of TGF-β1 concentration in lens epithelium of mice.

### TGF-β1 and TGF-βR1/2 inhibitor treatment

Recombinant human TGF-β1 (240-b-002, R&D Systems) was firstly added to the cell culture media with concentration gradient (0, 5,10, 20, 40, 80 ng /ml) to find the optimal concentration. In further stimulation experiment, 5 ng/ml of TGF-β1 was applied to LECs with a duration of 24 h. To treat primary mouse LECs, recombinant mouse TGF-β1 protein (7666-MB-005, R&D Systems) was used (5 ng/ml, 24 h).

To interfere with the TGF-β1 signaling, a selective TGF-βR1/2 inhibitor LY2109761 (S2704, Selleck) was used to treat primary mouse LECs with a working concentration of 10 μM for 24 h.

### Plasmid and siRNA transfection

Lipofectamine^TM^ 3000 Reagent (ThermoFisher Scientific) was used for plasmid and siRNA transfection and cells were incubated for 24 h before further analyses. To overexpress MAF, the cDNA sequence was cloned into pcDNA 3.1 vector to generate the overexpression construct. To knock down the expression of *Maf*, three siRNAs of the gene were designed and tested. After a qPCR assessment, one siRNA was selected for further cell treatment.

### Dual-luciferase reporter assay

To investigate the influence of SMAD2, SMAD3 and SMAD4 on promoter activity of *CRYBB1*, *CRYGD*, and *CRYBA2*, we cloned the putative promoters of the three crystallin genes respectively upstream of the pGL3-luciferase vector and compared transcription between cells with and without *SMAD* plasmid co-transfection. At the same time, to investigate the influence of MAF on promoter activity of *TGFB1*, we cloned the putative promoters of *TGFB1* (−1989/+211 region relative to the known transcription start site) upstream of the pGL3-luciferase vector and compared the transcription between cells with and without *MAF* plasmid co-transfection. After the verification of sequence syntheses, dual-luciferase reporter assay was conducted using luciferase assay kit (#E1500, Promega, USA) in accordance with the manufacturer’s instructions. 293T cell line was used as host cells in this experiment.

### Evaluation of cell proliferation

In evaluation of cell migration, LECs in the same region were photographed at the same time every day since the culture started till the 3rd day. Migration distance from the original rim of the lens epithelium was measured. EdU incorporation assay, cell counting kit-8, and Ki67 staining were conducted to evaluate cell proliferation and cell viability, all in accordance with the manufacturer’s instructions.

### Statistics

Statistical analyses were conducted using Prism 8.0 (GraphPad Software, Inc., USA). Results represent the mean ± standard deviation (SD) where applicable. Two-sample continuous variables that followed normal distribution (tested using Kruskal–Wallis test) were analyzed using two-tailed Student’s *t* test, and were analyzed using Mann–Whitney U test otherwise. For comparisons between paired samples, paired *t* test was applied. For comparisons between over two variables, one-way ANOVA and Tukey’s multiple comparisons test were conducted. Pearson correlation analyses were conducted to examine correlations between variables. Among variables based on repeated observations, repeated measures ANOVA was used. A *p*-value of less than 0.05 was considered statistically significant.

### Reporting summary

Further information on research design is available in the Nature Research Reporting Summary linked to this article.

## Supplementary information

Supplementary Information

Reporting Summary

## Data Availability

Human microarray data have been deposited in the Gene Expression Omnibus (GEO) under the accession code GSE136701. Online Jaspar database (http://jaspar.genereg.net/) was used for the prediction of MAF binding sites in promoters of crystallin genes. Source data are provided with this paper.

## Source data

Source Data
