# Author Correction: Aberrant TGF-β1 signaling activation by MAF underlies pathological lens growth in high myopia

**DOI:** 10.1038/s41467-022-35562-1

**Published:** 2022-12-20

**Authors:** Xiangjia Zhu, Yu Du, Dan Li, Jie Xu, Qingfeng Wu, Wenwen He, Keke Zhang, Jie Zhu, Linying Guo, Ming Qi, Ailin Liu, Jiao Qi, Guangyu Wang, Jiaqi Meng, Zhenglin Yang, Kang Zhang, Yi Lu

**Affiliations:** 1 Eye Institute, Eye & ENT Hospital, Fudan University, Shanghai, China; 2 NHC Key Laboratory of Myopia (Fudan University); Key Laboratory of Myopia, Chinese Academy of Medical Sciences, Shanghai, China; 3 Shanghai Key Laboratory of Visual Impairment and Restoration, Shanghai, China; 4 Beijing Institute of Ophthalmology, Beijing Tongren Hospital, Capital Medical University, Beijing, China; 5 Center for Biomedicine and Innovations, Faculty of Medicine, Macau University of Science and Technology and University Hospital, Macau, China; 6 Guangzhou KangRui Biological Pharmaceutical Technology Company, Guangzhou, China; 7 Institute of Genetics and Developmental Biology, Chinese Academy of Sciences, Beijing, China; 8 Guangzhou Women and Children’s Medical Center, Guangzhou Medical University, Guangzhou, China; 9 Department of Radiology, Eye & ENT Hospital, Fudan University, Shanghai, China; 10 Center for Biomedical Imaging, Fudan University, Shanghai, China; 11 State Key Laboratory of Medical Neurobiology and MOE Frontiers Center for Brain Science, Fudan University, Shanghai, China; 12 Sichuan Provincial Key Laboratory for Human Disease Gene Study and the Center for Medical Genetics, Department of Laboratory Medicine, Sichuan Academy of Medical Sciences & Sichuan Provincial People’s Hospital, University of Electronic Science and Technology, Chengdu, China; 13 Research Unit for Blindness Prevention of Chinese Academy of Medical Sciences (2019RU026), Sichuan Academy of Medical Sciences & Sichuan Provincial People’s Hospital, Chengdu, Sichuan China; 14 Guangzhou HuiBoRui Biological Pharmaceutical Technology Co., Ltd, Guangzhou, China

**Keywords:** Hereditary eye disease, Lens diseases, Refractive errors, Retinal diseases

Correction to: *Nature Communications* 10.1038/s41467-021-22041-2, published online 08 April 2021

In this article, the grant numbers 81790643 and 82121003 relating to the National Natural Science Foundation of the People’s Republic of China were omitted.

The funding from Sichuan Science and Technology Program and the CAMS Innovation Fund for Medical Sciences was also omitted.

In addition, the affiliation details for Zhenglin Yang were incorrectly given as ‘Sichuan Provincial Key Laboratory for Human Disease Gene Study, Sichuan Provincial People’s Hospital, University of Electronic Science and Technology of China, Chengdu, Sichuan, China’ but should have been ‘Sichuan Provincial Key Laboratory for Human Disease Gene Study and the Center for Medical Genetics, Department of Laboratory Medicine, Sichuan Academy of Medical Sciences & Sichuan Provincial People’s Hospital, University of Electronic Science and Technology, Chengdu, China.’ and ‘Research Unit for Blindness Prevention of Chinese Academy of Medical Sciences (2019RU026), Sichuan Academy of Medical Sciences & Sichuan Provincial People’s Hospital, Chengdu, Sichuan, China.’

The original article has been corrected.

