# Supplementary material for: Aberrant TGF-β1 signaling activation by MAF underlies pathological lens growth in high myopia
Source: Nat Commun. 2021 Apr 8;12:2102. doi: 10.1038/s41467-021-22041-2 (PMC8032689; doi:10.1038/s41467-021-22041-2)
Supplement: Supplementary file 1 — Supplementary Information [file 41467_2021_22041_MOESM1_ESM.pdf]

Supplementary Figure 1. mRNA levels of other crystallins in lens epithelium and protein levels of crystallins in lens mass of highly myopic subjects.

(a) Examination of mRNA levels of other crystallin genes by qPCR in lens epithelium of highly myopic subjects (n= 4 vs. 4). (b) Examination of protein levels of crystallins in lens mass of highly myopic subjects by parallel reaction monitoring (left: target crystallins n= 5 vs. 5, right: other crystallins n= 3 vs. 3, CRYBB1 $p = 0.002$, CRYGD $p = 0.017$, CRYBA1 $p = 0.010$).

n= biological replicates. Pooled lens epithelial samples were used and one lens mass from one eye was used as one sample.

Results are presented as mean ± SD. Level of significance was detected using Student's *t*-test. $^{**}p < 0.01$ and $^{*}p < 0.05$. Source data are provided as a Source Data file.

a

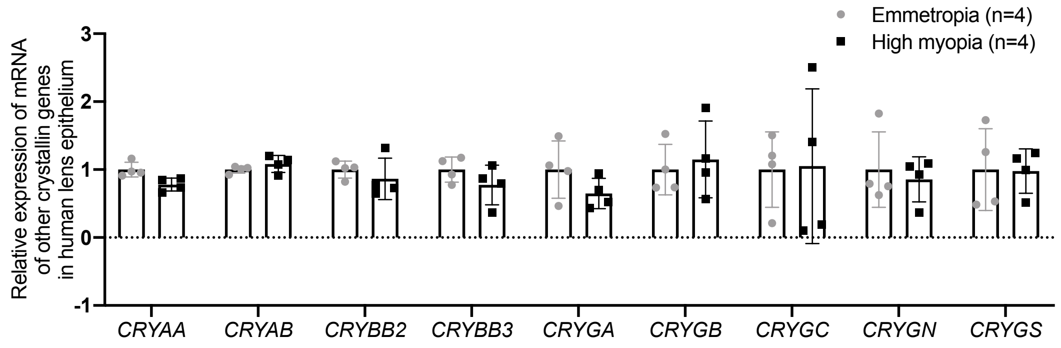

b

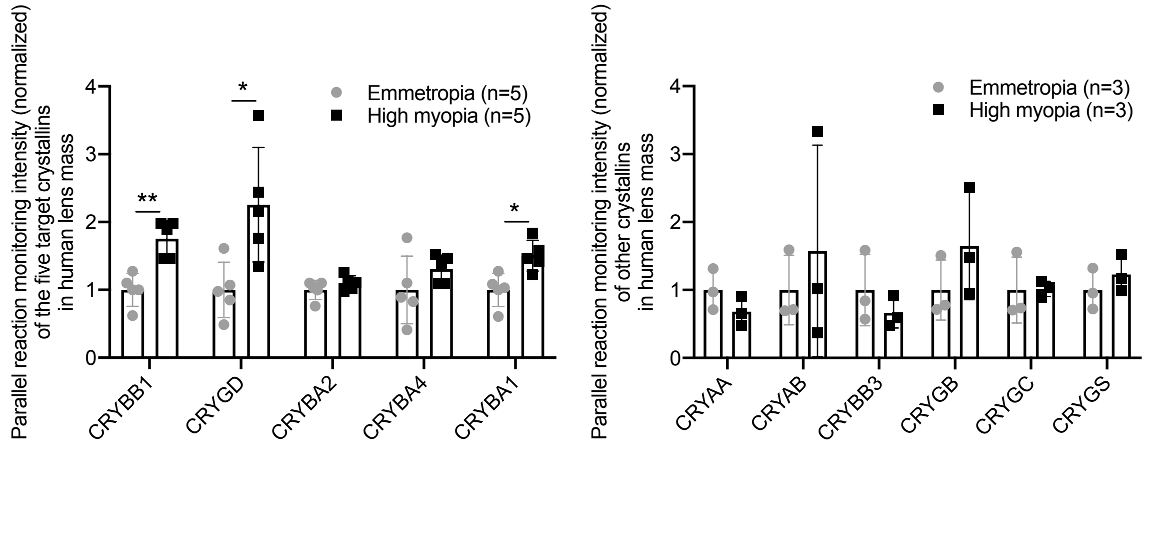

Supplementary Figure 2. Heat map from gene expression microarray analysis of crystallin genes in mouse lens epithelium.

(a) Gene expression microarray analysis of crystallin genes in lens epithelium of myopic vs. contralateral control lens in defocus-induced highly myopic mouse model (n=4 vs. 4). According to the list of 14 genes that human and mouse have in common, *Crybb1, Crygd, Cryba2, Cryba4, Crybb3, Cryga,* and *Crygs* were significantly up-regulated in myopic eyes.

(b) Gene expression microarray analysis of crystallin genes in lens epithelium of *Irbp* KO mice vs. wild type C57BL/6J mice (n = 3 vs. 4). According to the list of 14 genes that human and mouse have in common, *Crybb1, Crygd, Cryba4*, *Cryba1*, *Crybb2,* and *Cryga* were significantly up-regulated.

n= biological replicates. Pooled lens epithelial samples were used.

Source data are provided as a Source Data file.

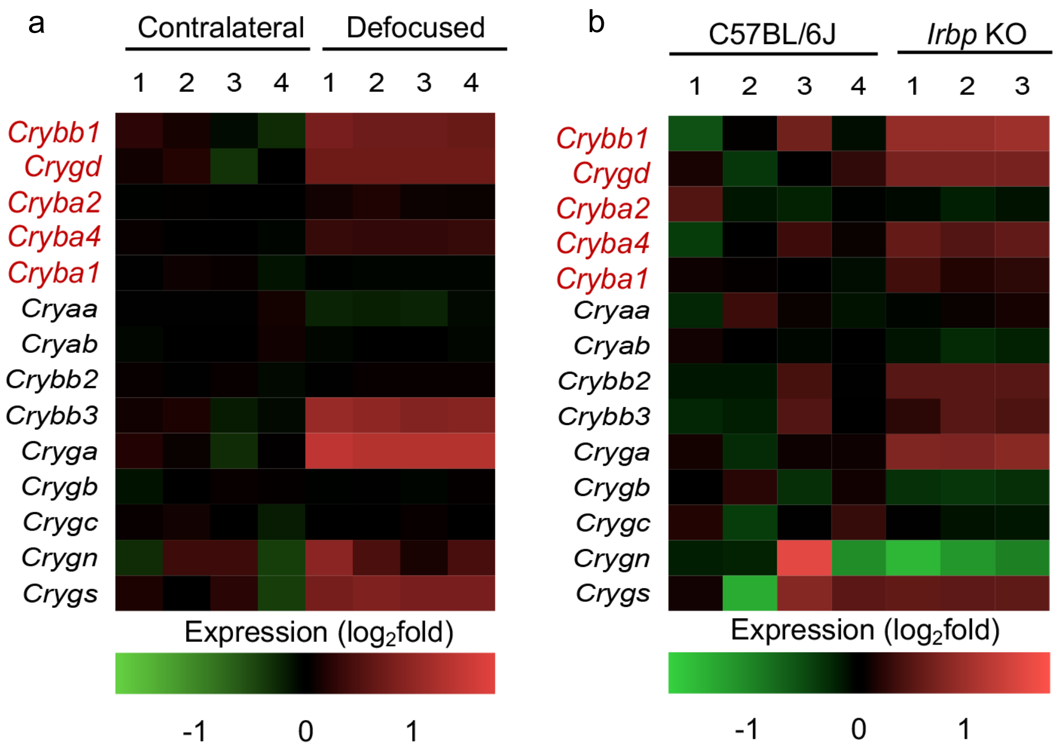

Supplementary Figure 3. Percentage contribution of different crystallins in total protein of normal human lens.

CRYBB1, CRYGD, CRYBA2, CRYBA4 and CRYBA1 together constituted around 28.0 ± 1.8% of the total protein in normal human lens (n= 3).

n=biological replicates. One lens from one eye was used as one sample.

Source data are provided as a Source Data file.

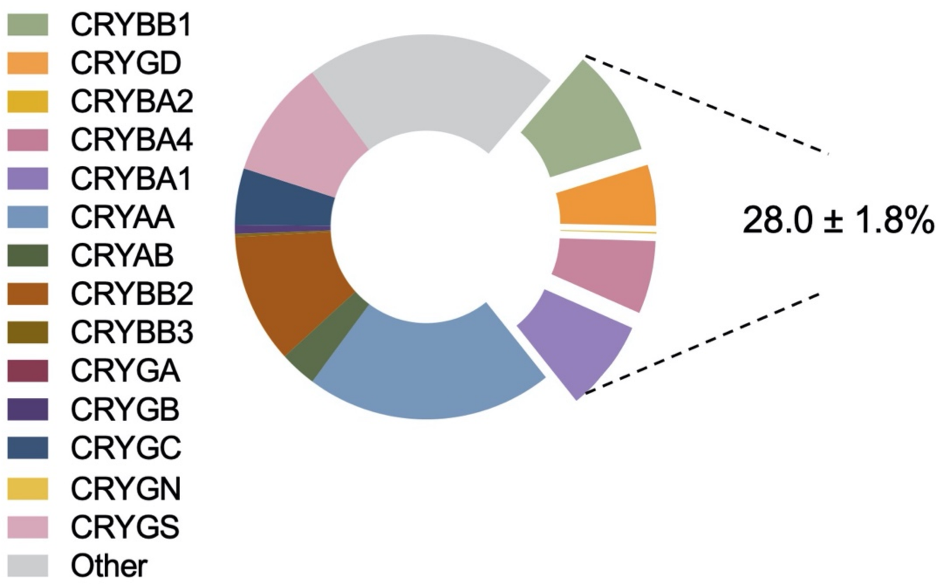

Supplementary Figure 4. The interface of 3D Slicer showing the calculation of lens volume.

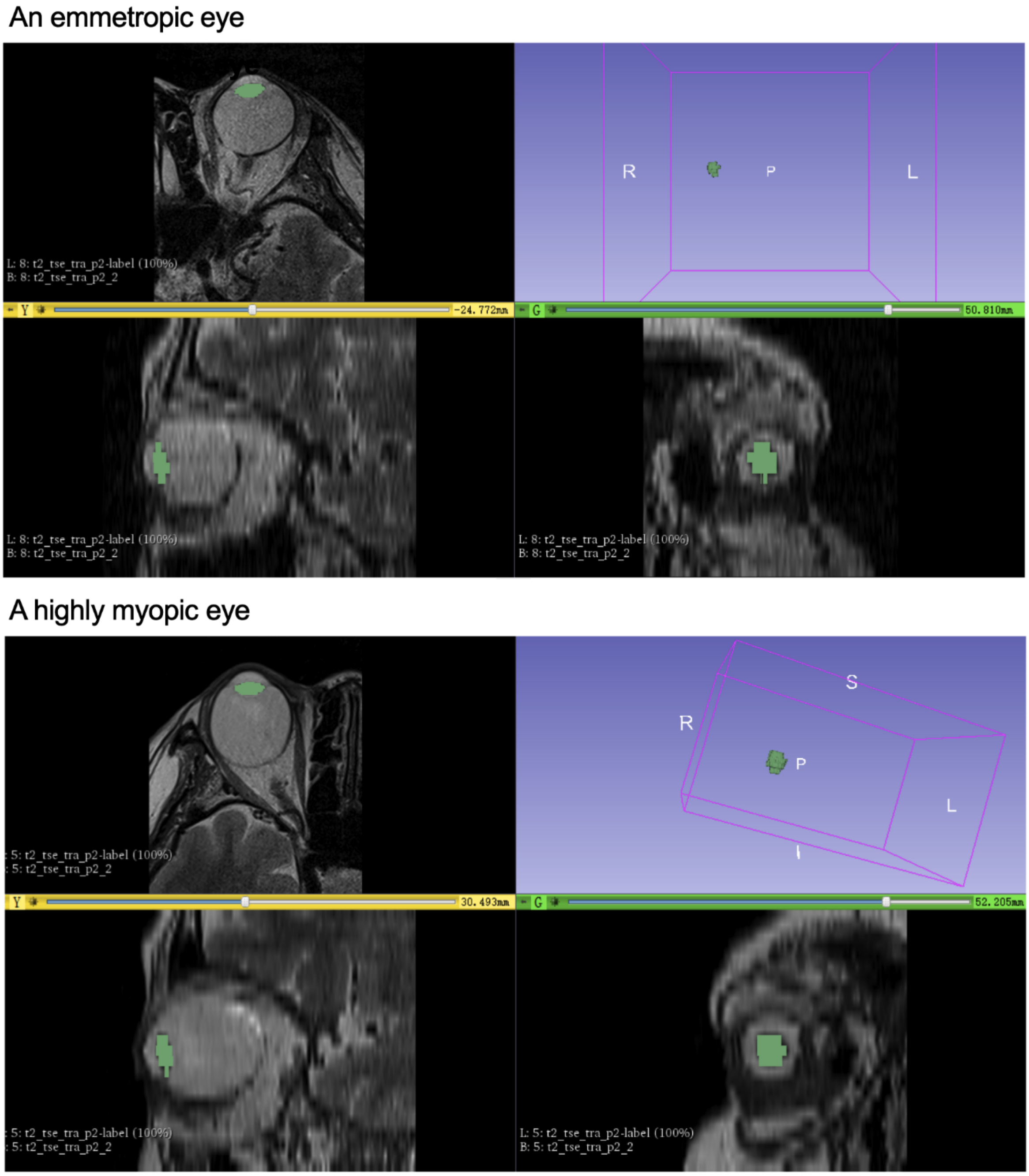

Supplementary Table S1. Primer sequences for crystallin genes in ChIP-qPCR.

Gene	Range	Forward Primer	Reverse Primer
CRYBB1	-400，-300	CAGAGGCCCCCCTAGGGGTAG	AGGGGAGACCCTGGGAG
CRYGD	-350，-250	GTTGCTTTTTTTGCTCCCCGA	TGGAAGTGGAGCTTCAG
CRYBA2	-1750，-1650	GCTTTGGCTTCCAAAGGTC	TATGGAAGCGGAGGGTCAAAG
CRYBA4	-1380，-1270	GAGACGGGGTCTTGCTCTGT	CTAGGGAGGCTGAGGCAGG
CRYBA1	-1856，-1756	ACGATTCCCCTACCTTACCTTCA	CTCACAGGCCCAAGTTACT

Supplementary Table S2. Primer sequences for qPCR.

Gene	Genome	Forward Primer	Reverse Primer
CRYBB1	Homo sapiens	CAGCGTGAAGGTCTCCAGTG	GACTGCATCTGTGGCTGGAA
CRYGD	Homo sapiens	CCTGTCTTCAGGACCGCTTC	CCCTGGCATCAGCAGGTACT
CRYBA2	Homo sapiens	GGGAGACTATCCTCGCTGGA	ACACGGCTGTCATTGTGGTT
CRYBA4	Homo sapiens	GGCTGACAATCTTCGAGCAA	GTGGACGTGGAAGGACCCTA
CRYBA1	Homo sapiens	GAAACCCTTCCAACCACCAA	GGAGCTGGTGAACTCCATCC
MAF	Homo sapiens	CAGCAAGGAGGAGGTGATCC	GCTGGTTCTTCTCCGACTCC
TGFB1	Homo sapiens	ATGACAAGTTCAAGCAGAG	CACTTGCAGTGTGTTATCC
CRYAA	Homo sapiens	CCTGCTGCCCTTCCTGTCGT	TCCTGGCGCTCGTTGTGCT
CRYAB	Homo sapiens	CCAGGATGAACATGGTTTCATC	ACAGGGATGAAGTAATGGTGAG
CRYBB2	Homo sapiens	TCATAGATGACGATGTACCCAG	TGTCCTTGTAGTCTCCCTTCTC
CRYBB3	Homo sapiens	ACGAACTAGAGAACTTCCAAGG	GATAATCCCCCTTCTCCAGAAC
CRYGA	Homo sapiens	GCGGCTGCTGGATGCTCTATG	AGGATTGGACCGAGTCGCTGAG
CRYGB	Homo sapiens	CCCAACCTACAACCCTATTTCA	CCCTCAATTCATCTCTGTCGTA
CRYGC	Homo sapiens	CCAAGGTCAACAATACTTGCTG	CTTCACTCAGCTCCATCATGAG
CRYGN	Homo sapiens	CTGCTTCAATCACCCCGACTTCC	CATGTGGTCACTGTGGCTGTTCC

CRYGS	Homo sapiens	TTGCTGGGTACATGTACATCTT	TTCGGTGGTTTCATACATCTGA
ACTB1	Homo sapiens	TTGTTACAGGAAGTCCCTTGCC	ATGCTATCACCTCCCCTGTGTG
Crybb1	Mus musculus	GTCTTCGAGCAGGAAAACTTTC	CTTCTCCAGGACAAACATCTCT
Crygd	Mus musculus	GATGGGTTTCAGTGACTCTGTC	TATCATCTGGCCTCTGTACTCT
Cryba2	Mus musculus	GGACAGCAGTTCATTCTAGAGA	CTGAAGGACAGCAATTGGTTG
Cryba4	Mus musculus	CTTCCAAGGACAGCAATATGTG	GAGAGGGATAGTCATCGTTCAG
Cryba1	Mus musculus	ATGGGTTGGTTCAACAATGAAG	GTATTCGGCGAATTGATTGGAT
Maf	Mus musculus	GCACTTCGACGACCGCTTCTC	TCGGATCACCTCCTCCTTGCTG
Tgfb1	Mus musculus	CCAGATCCTGTCCAAACTAAGG	CTCTTTAGCATAGTAGTCCGCT
Actb	Mus musculus	GGCAACGAGCGGTTCCGATG	CAGCACTGTGTTGGCATAGAGGTC

Supplementary Table S3. Antibody list.

Protein	Brand	Catalog number	Dilution
CRYBB1	proteintech, USA	60273-1-Ig	1:500
CRYGD	Novus, China	H0000142-M04	1:500
CRYBA2	proteintech, USA	15750-1-AP	1:2000
CRYBA4	Novus, China	NBP1-32741	1:500
CRYBA1	Novus, China	NBP1-33010	1:2000
MAF	Abcam,UK	ab77071	1:500
TGF-β1	Abcam,UK	ab92486	1:1000
TGF-βRI	Bioss, China	bs-0638R	1:500
Smad2/3	Abcam,UK	ab202445	1:1000
p-Smad2/3	Abcam,UK	ab63399	1:500
Smad4	Abcam,UK	ab40759	1:2000
β-Actin	Weiao, China	WB2196	1:1500
HRP-labeled Goat Anti-Rabbit IgG(H+L)	Beyotime, China	A0208	1:1000
HRP-labeled Goat Anti-Mouse IgG(H+L)	Beyotime, China	A0216	1:1000
Anti-rabbit, Secondary Antibody, Alexa Fluor 488	Thermo Fisher Scientific, USA	A32790	1:500
Anti-mouse, Secondary Antibody, Alexa Fluor 488	Thermo Fisher Scientific, USA	A-11001	1:1000
Rhodamine WGA	Vector Laboratories, USA	ZD0510	1:1000
Hoechst	Beyotime, China	C1011	1:2000

Supplementary Table S4. Peptide sequences for crystallins in parallel reaction monitoring.

Protein	Species	Peptide sequence
CRYBB1	Homo Sapiens	QWHLEGSFPVLATEPPK
CRYGD	Homo Sapiens	FNEIHSLNVLEGSWVLYELSNYR
CRYBA2	Homo Sapiens	GYQYVLER
CRYBA4	Homo Sapiens	EWGSHAPTFQVQSIR
CRYBA1	Homo Sapiens	ITIYDQENFQGK
CRYAA	Homo Sapiens	HFSPEDLTVK
CRYAB	Homo Sapiens	PAVTAAPK
CRYBB3	Homo Sapiens	HWNEWDASQPQLQSVR
CRYGB	Homo Sapiens	FLDWGAPNAK
CRYGC	Homo Sapiens	PNYQGQQYLLR
CRYGS	Homo Sapiens	VEGGTWAVYER
CRYBB1	Mus musculus	LIVFEQENFQGR
CRYGD	Mus musculus	GQMIEFTEDCPSLQDR
CRYBA2	Mus musculus	GYQYVLER
CRYBA4	Mus musculus	GFQYILESDHHSGDYK
CRYBA1	Mus musculus	ITIYDQENFQGK
