# Supplementary material for: Aberrant TGF-β1 signaling activation by MAF underlies pathological lens growth in high myopia
Source: Nat Commun. 2021 Apr 8;12:2102. doi: 10.1038/s41467-021-22041-2 (PMC8032689; doi:10.1038/s41467-021-22041-2)
Supplement: Supplementary file 2 — Reporting Summary [file 41467_2021_22041_MOESM2_ESM.pdf]

nature research

Corresponding author(s): Kang Zhang

Last updated by author(s): 12/27/2020

Reporting Summary

Nature Research wishes to improve the reproducibility of the work that we publish. This form provides structure for consistency and transparency in reporting. For further information on Nature Research policies, see Authors & Referees and the Editorial Policy Checklist .

Statistics

For all statistical analyses, confirm that the following items are present in the figure legend, table legend, main text, or Methods section.

n/a	Confirmed	
☐	☒	The exact sample size (*n*) for each experimental group/condition, given as a discrete number and unit of measurement
☐	☒	A statement on whether measurements were taken from distinct samples or whether the same sample was measured repeatedly
☐	☒	The statistical test(s) used AND whether they are one- or two-sided<br>*Only common tests should be described solely by name; describe more complex techniques in the Methods section.*
☐	☒	A description of all covariates tested
☐	☒	A description of any assumptions or corrections, such as tests of normality and adjustment for multiple comparisons
☐	☒	A full description of the statistical parameters including central tendency (e.g. means) or other basic estimates (e.g. regression coefficient) AND variation (e.g. standard deviation) or associated estimates of uncertainty (e.g. confidence intervals)
☐	☒	For null hypothesis testing, the test statistic (e.g. *F*, *t*, *r*) with confidence intervals, effect sizes, degrees of freedom and *P* value noted<br>*Give P values as exact values whenever suitable.*
☒	☐	For Bayesian analysis, information on the choice of priors and Markov chain Monte Carlo settings
☒	☐	For hierarchical and complex designs, identification of the appropriate level for tests and full reporting of outcomes
☐	☒	Estimates of effect sizes (e.g. Cohen's *d*, Pearson's *r*), indicating how they were calculated

Our web collection on statistics for biologists contains articles on many of the points above.

Software and code

Policy information about availability of computer code

Data collection	For MRI data collection, sample size was calculated based on results of preliminary investigation using G*Power Software 3.1.<br>Lens volume data were collected using an open-source software (3D Slicer 4.10.1 r27931, https://www.slicer.org/).<br>The axial length and the maximum cross-sectional area of lens of mice were measured with ParaVision ® 6.0.1 software (Bruker, USA).
Data analysis	Most statistical analyses were conducted using the Prism 8.0 (GraphPad Software, Inc., USA).<br>For microarray, differential gene expression was determined using the limma statistical package 3.26.9 as described at http://www.bioconductor.org.

For manuscripts utilizing custom algorithms or software that are central to the research but not yet described in published literature, software must be made available to editors/reviewers. We strongly encourage code deposition in a community repository (e.g. GitHub). See the Nature Research guidelines for submitting code & software for further information.

Data

Policy information about availability of data

All manuscripts must include a data availability statement. This statement should provide the following information, where applicable:

- Accession codes, unique identifiers, or web links for publicly available datasets
- A list of figures that have associated raw data
- A description of any restrictions on data availability

Human microarray data have been deposited in the Gene Expression Omnibus (GEO) under the accession code GSE136701.
Online Jaspar database (http://jaspar.genereg.net/) was used for prediction of MAF binding sites in promoters of crystallin genes.
The source data underlying figures shown in the manuscript and the raw images for the immunoblots are provided as a Source Data file.

Field-specific reporting

Please select the one below that is the best fit for your research. If you are not sure, read the appropriate sections before making your selection.

☒ Life sciences ☐ Behavioural & social sciences ☐ Ecological, evolutionary & environmental sciences

For a reference copy of the document with all sections, see nature.com/documents/nr-reporting-summary-flat.pdf

Life sciences study design

All studies must disclose on these points even when the disclosure is negative.

Sample size

For MRI data collection, sample size was calculated based on results of preliminary investigation using G*Power Software 3.1 with a desired significance level of 5% and test power of 0.95. For other experiments, sample sizes were determined according to experience with previous experiments. References were as follows:
Zhu, X., Li, D., Du, Y., He, W. & Lu, Y. DNA hypermethylation-mediated downregulation of antioxidant genes contributes to the early onset of cataracts in highly myopic eyes. Redox biology 19, 179-189 (2018).
Jonathan, M.F. et al. Lysosomal protease deficiency or substrate overload induces an oxidative-stress mediated STAT3-dependent pathway of lysosomal homeostasis. Nat Commun 8, 5343 (2018).
Sample sizes are shown in each figure legend.

Data exclusions

No data were excluded.

Replication

At least three replications were made in all experiments. And all attempts at replication were successful.

Randomization

No randomization of mice. For establishement of defocus-induced myopic mouse model, male C57BL/6J mice were subjected to continuous defocus all to the right eye. Wild-type mice and Irbp KO mice in comparison were age- and sex-matched.
For experiments using specimens with certain treatment, our samples were consecutively collected and randomly picked when deciding which one to use in a specific assay or to form a pooled sample or to be treated.

Blinding

For MRI data collection, researchers did not know the diagnosis of high myopia or emmetropia before measurement, however axial length and lens dimensions were measured simultaneously in the same image, thus blinding could not be done here. As for experiments included in this study, most of them contained several steps, including sample collection, treatment, establishment of mouse model, measurement of refraction, etc., investigators had to keep careful track of the conditions and steps. Thus, it would be exceedingly difficult to blind such studies and investigators were not blinded, however our results were validated in biological replicates.

Reporting for specific materials, systems and methods

We require information from authors about some types of materials, experimental systems and methods used in many studies. Here, indicate whether each material, system or method listed is relevant to your study. If you are not sure if a list item applies to your research, read the appropriate section before selecting a response.

Materials & experimental systems

n/a	Involved in the study	
☐	☒	Antibodies
☐	☒	Eukaryotic cell lines
☒	☐	Palaeontology
☐	☒	Animals and other organisms
☐	☒	Human research participants
☒	☐	Clinical data

Methods

n/a	Involved in the study	
☒	☐	ChIP-seq
☒	☐	Flow cytometry
☒	☐	MRI-based neuroimaging

Antibodies

Antibodies used

Protein Brand Catalog number
CRYBB1 proteintech, USA 60273-1-Ig dilution: 1:500
CRYGD Novus, China H0000142-M04 dilution:1:500
CRYBA2 proteintech, USA 15750-1-AP dilution:1:2000
CRYBA4 Novus, China NBP1-32741 dilution:1:500
CRYBA1 Novus, China NBP1-33010 dilution: 1:2000
MAF Abcam,UK ab77071 dilution: 1:500
TGF-β1 Abcam,UK ab92486 dilution: 1:1000
TGF-βRI Bioss, China bs-0638R dilution: 1:500
Smad2/3 Abcam,UK ab202445 dilution: 1:1000

p-Smad2/3 Abcam,UK ab63399 dilution: 1:500
Smad4 Abcam,UK ab40759 dilution: 1:2000
β-Actin Weiao, China WB2196 dilution: 1:1500
HRP-labeled Goat Anti-Rabbit IgG(H+L) Beyotime, China A0208 dilution: 1:1000
HRP-labeled Goat Anti-Mouse IgG(H+L) Beyotime, China A0216 dilution: 1:1000
Anti-rabbit, Secondary Antibody,
Alexa Fluor 488 Thermo Fisher Scientific, USA A32790 dilution: 1:500
Anti-mouse, Secondary Antibody,
Alexa Fluor 488 Thermo Fisher Scientific, USA A-11001 dilution: 1:1000
Rhodamine WGA Vector Laboratories, USA ZD0510 dilution: 1:1000
Hoechst Beyotime, China C1011 dilution: 1:2000

Validation

Primary antibodies for detection of CRYBB1, CRYBA2, CRYBA4, CRYBA1, MAF, TGF-βRI, Smad2/3, p-Smad2/3 and Smad 4 were all validated by the supplier with blots shown on the manufacturer's websites and the usages of them for testing target protein expression in either human or mouse samples of our study were all accordant with Species Reactivity specified in manufacturer's instructions. As for the primary antibodies for detection of CRYGD and TGF-β1, their reactivity with human tissue was stated on the manufacturer's website. Before we used them for formal assay using mouse tissue, validation of the antibody was conducted by us by checking the reactivity and the band position in gel blots.

Eukaryotic cell lines

Policy information about cell lines

Cell line source(s)

The 293T cell line used in Luciferase was obtained from the Cell Bank, Chinese Academy of Sciences.

Authentication

The cell line was authenticated using short tandem repeat profiling (Shanghai Biowing Applied Biotechnology, China).

Mycoplasma contamination

The cell line was tested negative for mycoplasma contamination.

Commonly misidentified lines
(See ICLAC register)

No commonly misidentified lines.

Animals and other organisms

Policy information about studies involving animals; ARRIVE guidelines recommended for reporting animal research

Laboratory animals

3-4-week male C57BL/6J mice were used for establishment of myopic model and were sacrificed at 8 week for further analyses.
3-4-week male C57BL/6J mice were also used for procurement of mouse lens epithelium for primary culture and further analyses.
8-week male Irbp KO mice (#023080) were used for observation of lens dimensions and lens fiber compaction and examination of protein expressions in lens with 8-week male C57BL/6J mice being control.
Mice were bred and housed in clear cages and maintained at temperature of 21°C with 40-60% humidity with a 12:12-h light-dark cycle (light on at 7:00 AM and off at 7:00 PM).

Wild animals

No wild animals were used.

Field-collected samples

No field-collected samples were used.

Ethics oversight

Animal experiments were approved by the Ethics Committee for Animal Studies of Eye & ENT Hospital of Fudan University and experimental procedures all conformed to the ARVO Statement for the use of animals in research.

Note that full information on the approval of the study protocol must also be provided in the manuscript.

Human research participants

Policy information about studies involving human research participants

Population characteristics	The study included highly myopic and emmetropic population.<br>In magnetic resonance imaging (MRI) section, lens dimensions were measured using images retrieved from the digital archiving system of our hospital, including 105 highly myopic eyes and 144 emmetropic eyes. The two groups were comparable in age (49.36 ± 14.49 yrs. in the highly myopic group vs. 50.63 ± 14.33 yrs. in the emmetropic control group). Age ranges were 24~76 yrs. and 25~76 yrs. respectively. Gender composition was 42.9 % male in highly myopic group vs. 45.1% male in the emmetropic control group.<br>As to the MRI data for correlation analysis, totally 48 highly myopic patients were recruited. Age ranged from 37~58 yrs. and was averagely 49.06 ±5.96 yrs. Gender composition was 41.7% male.<br>With regards to the patients that we recruited for collection of biosamples, age and gender distributions were also comparable. In the highly myopic group, the mean age was 49.53 ± 6.58 yrs. and the age range was 35~61 yrs. In the emmetropic control group, the mean age was 50.06 ± 4.31 yrs. and the age range was 40~63 yrs. The gender composition was 51.2% male in highly myopic group vs. 58.8% male in the emmetropic control group.
Recruitment	Subjects were recruited at Eye & ENT Hospital of Fudan University. Those who met the including criteria were asked to participate in our study as described in the manuscript. Participation was totally voluntary.<br>Since clinical data and biospecimens were consecutively collected from all the eligible patients in our hospital, we do not think there is a potential self-selection bias or other biases that may be present and are likely to impact results.
Ethics oversight	The study was approved by the Ethics Committee of the Eye & ENT Hospital of Fudan University, Shanghai, China.

Note that full information on the approval of the study protocol must also be provided in the manuscript.
